# Species delimitation of crab-eating frogs (*Fejervarya cancrivora* complex) clarifies taxonomy and geographic distributions in mainland Southeast Asia

**DOI:** 10.3897/zookeys.883.37544

**Published:** 2019-10-28

**Authors:** Siriporn Yodthong, Bryan L. Stuart, Anchalee Aowphol

**Affiliations:** 1 Department of Zoology, Faculty of Science, Kasetsart University, Bangkok, Thailand Kasetsart University Bangkok Thailand; 2 Section of Research & Collections, North Carolina Museum of Natural Sciences, Raleigh, NC, USA North Carolina Museum of Natural Sciences Raleigh United States of America

**Keywords:** Amphibia, cryptic species, Dicroglossidae, systematics, taxonomy

## Abstract

The taxonomy and geographic distributions of species of crab-eating frogs (*Fejervarya cancrivora* complex) in mainland Southeast Asia have been highly uncertain. Three taxonomic names are used in recent literature (*F. cancrivora*, *F. raja*, and *F. moodiei*) but the applications of these names to localities has been inconsistent, especially owing to the lack of available molecular data for *F. raja*. Morphometric and mitochondrial DNA variation was examined in these frogs, including name-bearing types and topotypes of all three species. Findings corroborate evidence for the existence of two species in coastal mainland Southeast Asia, with *F. moodiei* having a wide geographic distribution and *F. cancrivora* sensu stricto occurring only in extreme southern Thailand and peninsular Malaysia. *Fejervarya raja* is shown to be only a large-bodied population of *F. cancrivora* sensu stricto and is synonymized with that species. Revised descriptions of *F. moodiei* and *F. cancrivora* sensu stricto are provided.

## Introduction

Southeast Asia harbors high levels of amphibian species diversity and endemism (Brown and Stuart 2012), and new species continue to be discovered and described (e.g., Geissler et al. 2014; Phimmachak et al. 2015; Sheridan and Stuart 2018). Moreover, recent evaluations of morphological and molecular diversity of Southeast Asian amphibians have routinely shown that long-recognized geographically widespread single species actually represent complexes of cryptic species (Stuart et al. 2006b; Aowphol et al. 2013; Phimmachak et al. 2015; Sheridan and Stuart 2018). The presence of cryptic species in Southeast Asian amphibians has hindered accurately assessing species boundaries and, ultimately, efforts to conserve them (Bickford et al. 2006; Sheridan and Stuart 2018). Even geographically widespread, human commensalist species may contain unrecognized diversity that alters their priority for conservation (Wogan et al. 2016).

Species of frogs in the genus *Fejervarya* Bolkay, 1915 have been subject to numerous investigations into cryptic diversity in efforts to resolve species boundaries and uncertain taxonomy in South, Southeast and East Asia (e.g., Vieth et al. 2001; Matsui et al. 2007; Islam et al. 2008; Kotaki et al. 2010; Sanchez et al. 2018). A notable challenge remains with the crab-eating frog, *F. cancrivora* (Gravenhorst, 1829), a species that is remarkable in its ability to thrive in brackish or salt water (e.g., Gordon et al. 1961; Balinsky et al. 1972; Wright et al. 2004; Hopkins and Brodie 2015). *Fejervarya cancrivora* occurs in coastal areas throughout much of Southeast Asia, and as expected owing to its large geographic range, recent molecular investigations have hypothesized the existence of cryptic species and discordance between taxonomy and species diversity within the taxon (Kurniawan et al. 2010, 2011). Historically, the name *F. cancrivora* had been erroneously applied to larger members of the *F. limnocharis* complex, but application of the name was stabilized following designation of a neotype specimen from Cianjur, West Java, Indonesia, by Dubois and Ohler (2000). Taylor (1920) described the Philippine populations of *F. cancrivora* as a distinct species, *F. moodiei* (originally *Rana moodiei* Taylor, 1920) based on an adult female collected at Manila, Luzon, Philippines. Smith (1930) described a population of *F. cancrivora* specimens having large body sizes from Pattani, Thailand, as *F. raja* (originally *R. cancrivora raja* Smith, 1930).

Two of these species, *F. cancrivora* and *F. raja*, have been reported from Thailand, where they occur in the vicinity of sea shores or river mouths (Smith 1930; Taylor 1962; Nutphund 2001; Chan-ard 2003; Chuaynkern and Chuaynkern 2012). However, these designations have been uncertain. Iskandar (1998) suggested that *F. raja* from Thailand might just be unusually large individuals of *F. cancrivora*. Other authors have questioned the distinctiveness of the Philippine *F. moodiei* from *F. cancrivora*, and have synonymized them (Smith 1927; Inger 1954) or considered *F. moodiei* to be invalid (Matsui et al. 2007). Analyses of morphological and molecular variation, as well as laboratory crossing experiments, revealed three distinct “types” (= forms) of *F. cancrivora* across its large geographic range: a large type considered to be true *F. cancrivora*, a mangrove type considered to be *F. moodiei*, and a Sulawesi type that might belong to an undescribed species (Kurniawan et al. 2010, 2011). Their results also inferred that *F. raja* might be conspecific with *F. cancrivora*. A lack of molecular data from true *F. raja* and examination of type specimens in the *F. cancrivora* complex (Islam et al. 2008; Kurniawan et al. 2010, 2011) have hindered resolving species boundaries and taxonomy within the crab-eating frogs.

In this study, we examined morphology and mitochondrial DNA variation in historical and newly-collected museum specimens of the *F. cancrivora* complex from Thailand and adjacent Asian countries to evaluate and clarify the taxonomic status of *F. cancrivora*, *F. moodiei* and *F. raja*. Importantly, our analyses included molecular and morphological data of topotypes of *F. raja*, and morphological data from the name-bearing type specimens of *F. cancrivora* and *F. moodiei*.

## Materials and methods

### Sampling

**Figure 1.**
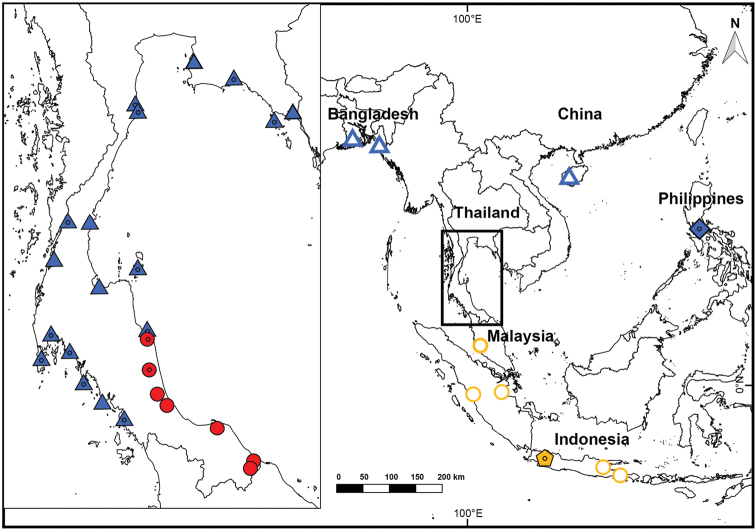
Map of sampling localities of the *Fejervarya cancrivora* complex, including *F. cancrivora* neotype (yellow pentagon), *F. cancrivora* sensu stricto (yellow circles), *F. moodiei* holotype (blue diamond), *F. moodiei* (blue triangles), and *F. cancrivora* samples that were referred to *F. raja* (red circles) prior to this study. Open symbols indicate molecular data only, shaded symbols indicate morphological data only, and shaded symbols with center dots indicate both molecular and morphological data were studied.

During 2015–2017, specimens of *F. cancrivora* were collected at 12 localities and *F. raja* at two localities in Thailand (Fig. 1). Specimens were humanely euthanized using tricainemethanesulfonate (MS222) solution. Liver or muscle tissue was removed from each individual, preserved in 95% ethyl alcohol, and stored at -20 °C for molecular analysis. Voucher specimens were initially fixed in 10% buffered formalin and later transferred to 70% ethyl alcohol for long-term preservation. Tissue samples and voucher specimens were deposited in the herpetological collection of the Zoological Museum, Kasetsart University, Bangkok, Thailand (**ZMKU**). Comparative material was also studied in the holdings of ZMKU, Carnegie Museum of Natural History (**CM**), Field Museum of Natural History [**FMNH**; formerly Chicago Natural History Museum (**CNHM**)], and Thailand Natural History Museum (**THNHM**; Table 1; Appendix 1).

**Table 1.** Specimens of *Fejervarya* used in (A) molecular and/or (B) morphological analyses.

Species identification		Locality	Museum No.	GenBank Acession No.	Type of analyses	Reference
Previous study	This study					
*F. moodiei* (holotype)	*F. moodiei*	Manila, Luzon, Philippines	CM 3724	–	B	This study
*F. cancrivora*	*F. moodiei*	Malaysia	CNHM 161312	–	B	This study
*F. cancrivora*	*F. moodiei*	Northern Luzon	FMNH 161693	–	B	This study
*F. cancrivora*	*F. moodiei*	Northern Luzon	FMNH 161697	–	B	This study
*F. cancrivora*	*F. moodiei*	Chonburi, Thailand	FMNH 190532	–	B	This study
*F. cancrivora*	*F. moodiei*	Mueang Surat Thani, Surat Thani, Thailand	THNHM 05857	–	B	This study
*F. cancrivora*	*F. moodiei*	Moo Ko Chumphon National Park, Chumphon, Thailand	THNHM 01032	–	B	This study
*F. cancrivora*	*F. moodiei*	Moo Ko Chumphon National Park, Chumphon, Thailand	THNHM 01031	–	B	This study
*F. cancrivora*	*F. moodiei*	Moo Ko Chumphon National Park, Chumphon, Thailand	THNHM 01033	–	B	This study
*F. cancrivora*	*F. moodiei*	Ko Libong, Trang, Thailand	THNHM 02249	–	B	This study
*F. cancrivora*	*F. moodiei*	Songkhla lake, Songkhla, Thailand	THNHM 02405	–	B	This study
*F. cancrivora*	*F. moodiei*	Songkhla lake, Phatthalung, Thailand	THNHM 04332	–	B	This study
*F. cancrivora*	*F. moodiei*	Kleang, Rayong, Thailand	THNHM 14252	–	B	This study
*F. cancrivora*	*F. moodiei*	Kleang, Rayong, Thailand	THNHM 14254	–	B	This study
*F. cancrivora*	*F. moodiei*	Kleang, Rayong, Thailand	THNHM 14255	–	B	This study
*F. cancrivora*	*F. moodiei*	Kleang, Rayong, Thailand	THNHM 14256	–	B	This study
*F. cancrivora*	*F. moodiei*	Mueang Trat, Trat, Thailand	THNHM 16631	–	B	This study
*F. cancrivora*	*F. moodiei*	Tak Bai, Narathiwat, Thailand	THNHM 19720	–	B	This study
*F. cancrivora*	*F. moodiei*	Tak Bai, Narathiwat, Thailand	THNHM 19721	–	B	This study
*F. cancrivora*	*F. moodiei*	Tak Bai, Narathiwat, Thailand	THNHM 19724	–	B	This study
*F. cancrivora*	*F. moodiei*	Tak Bai, Narathiwat, Thailand	THNHM 19725	–	B	This study
*F. cancrivora*	*F. moodiei*	Suk Samran, Ranong, Thailand	THNHM 25736	–	B	This study
*F. cancrivora*	*F. moodiei*	Suk Samran, Ranong, Thailand	THNHM 26002	–	B	This study
*F. cancrivora*	*F. moodiei*	Suk Samran, Ranong, Thailand	THNHM 26016	–	B	This study
*F. cancrivora*	*F. moodiei*	Sam Roi Yot, Prachuap Khiri Khan, Thailand	ZMKU AM 01368	MN453492	A	This study
*F. cancrivora*	*F. moodiei*	Sam Roi Yot, Prachuap Khiri Khan, Thailand	ZMKU AM 01369	MN453493	A, B	This study
*F. cancrivora*	*F. moodiei*	Sam Roi Yot, Prachuap Khiri Khan, Thailand	ZMKU AM 01370	MN453494	A	This study
*F. cancrivora*	*F. moodiei*	Sam Roi Yot, Prachuap Khiri Khan, Thailand	ZMKU AM 01371	–	B	This study
*F. cancrivora*	*F. moodiei*	Kraburi, Ranong, Thailand	ZMKU AM 01373	MN453495	A, B	This study
*F. cancrivora*	*F. moodiei*	Kraburi, Ranong, Thailand	ZMKU AM 01375	MN453496	A, B	This study
*F. cancrivora*	*F. moodiei*	Mueang, Phuket, Thailand	ZMKU AM 01376	–	B	This study
*F. cancrivora*	*F. moodiei*	Mueang, Phuket, Thailand	ZMKU AM 01377	MN453497	A	This study
*F. cancrivora*	*F. moodiei*	Mueang, Phuket, Thailand	ZMKU AM 01381	MN453498	A, B	This study
*F. cancrivora*	*F. moodiei*	Ko Samui, Surat Thani, Thailand	ZMKU AM 01384	MN453499	A, B	This study
*F. cancrivora*	*F. moodiei*	Ko Samui, Surat Thani, Thailand	ZMKU AM 01386	–	B	This study
*F. cancrivora*	*F. moodiei*	Ko Samui, Surat Thani, Thailand	ZMKU AM 01387	MN453500	A, B	This study
*F. cancrivora*	*F. moodiei*	Mueang Phang-nga, Phang-nga, Thailand	ZMKU AM 01390	MN453501	A, B	This study
*F. cancrivora*	*F. moodiei*	Mueang Phang-nga, Phang-nga, Thailand	ZMKU AM 01394	MN453502	A, B	This study
*F. cancrivora*	*F. moodiei*	Mueang Phang-nga, Phang-nga, Thailand	ZMKU AM 01397	MN453503	A, B	This study
*F. cancrivora*	*F. moodiei*	Mueang Phang-nga, Phang-nga, Thailand	ZMKU AM 01398	–	B	This study
*F. cancrivora*	*F. moodiei*	Mueang Phuket, Phuket, Thailand	ZMKU AM 01399	MN453504	A, B	This study
*F. cancrivora*	*F. moodiei*	Mueang Phuket, Phuket, Thailand	ZMKU AM 01400	–	B	This study
*F. cancrivora*	*F. moodiei*	Mueang Phuket, Phuket, Thailand	ZMKU AM 01404	–	B	This study
*F. cancrivora*	*F. moodiei*	Ko Lanta, Krabi, Thailand	ZMKU AM 01405	MN453505	A, B	This study
*F. cancrivora*	*F. moodiei*	Ko Lanta, Krabi, Thailand	ZMKU AM 01407	–	B	This study
*F. cancrivora*	*F. moodiei*	Ko Lanta, Krabi, Thailand	ZMKU AM 01409	MN453506	A	This study
*F. cancrivora*	*F. moodiei*	Ko Lanta, Krabi, Thailand	ZMKU AM 01413	MN453507	A	This study
*F. cancrivora*	*F. moodiei*	Khanom, Nakhon Si Thammarat, Thailand	ZMKU AM 01436	–	B	This study
*F. cancrivora*	*F. moodiei*	Ko Chang, Trat, Thailand	ZMKU AM 01442	MN453508	A, B	This study
*F. cancrivora*	*F. moodiei*	Ko Chang, Trat, Thailand	ZMKU AM 01446	MN453509	A, B	This study
*F. cancrivora*	*F. moodiei*	Ko Chang, Trat, Thailand	ZMKU AM 01451	MN453510	A, B	This study
*F. cancrivora*	*F. moodiei*	Ko Chang, Trat, Thailand	ZMKU AM 01453	–	B	This study
*F. cancrivora*	*F. moodiei*	Pak Phanang, Nakhon Si Thammarat, Thailand	ZMKU AM 01467	MN453511	A, B	This study
*F. cancrivora*	*F. moodiei*	Pak Phanang, Nakhon Si Thammarat, Thailand	ZMKU AM 01469	–	B	This study
*F. cancrivora*	*F. moodiei*	Pak Phanang, Nakhon Si Thammarat, Thailand	ZMKU AM 01470	–	B	This study
*F. cancrivora*	*F. moodiei*	Pak Phanang, Nakhon Si Thammarat, Thailand	ZMKU AM 01475	MN453512	A, B	This study
*F. cancrivora*	*F. moodiei*	Pak Phanang, Nakhon Si Thammarat, Thailand	ZMKU AM 01479	MN453513	A, B	This study
*F. cancrivora*	*F. moodiei*	Kraburi, Ranong, Thailand	ZMKU AM 01485	MN453514	A, B	This study
*F. cancrivora*	*F. moodiei*	Kraburi, Ranong, Thailand	ZMKU AM 01486	–	B	This study
*F. cancrivora*	*F. moodiei*	Mueang Krabi, Krabi, Thailand	ZMKU AM 01488	–	B	This study
*F. cancrivora*	*F. moodiei*	Mueang Krabi, Krabi, Thailand	ZMKU AM 01489	–	B	This study
*F. cancrivora*	*F. moodiei*	Kui Buri, Prachuap Khiri Khan, Thailand	ZMKU AM 01492	–	B	This study
*F. cancrivora*	*F. moodiei*	La-ngu, Satun, Thailand	ZMKU AM 01493	MN453515	A, B	This study
*F. cancrivora*	*F. moodiei*	La-ngu, Satun, Thailand	ZMKU AM 01494	–	B	This study
*F. cancrivora*	*F. moodiei*	La-ngu, Satun, Thailand	ZMKU AM 01498	MN453516	A, B	This study
*F. cancrivora*	*F. moodiei*	La-ngu, Satun, Thailand	ZMKU AM 01503	MN453517	A, B	This study
*F. cancrivora*	*F. moodiei*	Kleang, Rayong, Thailand	ZMKU AM 01516	MN453518	A, B	This study
*F. cancrivora*	*F. moodiei*	Kleang, Rayong, Thailand	ZMKU AM 01520	MN453519	A, B	This study
*F. cancrivora*	*F. moodiei*	Manila, Philippines	–	AB070738	A	Sumida et al. (2002)
*F. cancrivora*	*F. moodiei*	Negros Island, Philippines	–	AF206473	A	Chen et al. (2005)
*F. cancrivora*	*F. moodiei*	Hainan, China	–	DQ458252	A	Che et al. (2007)
*F. moodiei*	*F. moodiei*	Dacope, Khulna, Bangladesh	–	AB530508	A	Hasan et al. (2012)
*F. moodiei*	*F. moodiei*	Teknaf, Cox’s Bazar, Bangladesh	–	AB543602	A	Hasan et al. (2012)
*F. cancrivora*	*F. cancrivora*	Cianjur, Java, Indonesia	–	AB444684	A	Kurniawan et al. (2010)
*F. cancrivora*	*F. cancrivora*	Padang, Sumatra, Indonesia	–	AB444685	A	Kurniawan et al. (2010)
*F. cancrivora*	*F. cancrivora*	Selangor, Malaysia	–	AB444688	A	Kurniawan et al. (2010)
*F. cancrivora*	*F. cancrivora*	Bogor, Java, Indonesia	–	AB444689	A	Kurniawan et al. (2010
*F. cancrivora*	*F. cancrivora*	Banyumas, Java, Indonesia	–	AB444690	A	Kurniawan et al. (2010)
*F. cancrivora*	*F. cancrivora*	Malang, East Java, Indonesia	–	AB570273	A	Kurniawan et al. (2014)
*F. cancrivora*	*F. cancrivora*	Denpasar, Bali, Indonesia	–	AB570277	A	Kurniawan et al. (2014)
*F. cancrivora* (neotype)	*F. cancrivora*	Cianjur, Java, Indonesia	FMNH 256688	–	B	This study
*F. cancrivora*	*F. cancrivora*	Java, Indonesia	CNHM 131093	–	B	This study
*F. cancrivora*	*F. cancrivora*	Java, Indonesia	CNHM 131100	–	B	This study
*F. cancrivora*	*F. cancrivora*	Java, Indonesia	CMNH 161102	–	B	This study
*F. cancrivora*	*F. cancrivora*	Java, Indonesia	CNHM 313095	–	B	This study
*F. cancrivora*	*F. cancrivora*	Java, Indonesia	FMNH 131108	–	B	This study
*F. cancrivora*	*F. cancrivora*	Java, Indonesia	FMNH 131111	–	B	This study
*F. raja*	*F. cancrivora*	Nakhon Si Thammarat, Thailand	FMNH 174052	–	B	This study
*F. raja*	*F. cancrivora*	Phatthalung, Thailand	FMNH 174053	–	B	This study
*F. raja*	*F. cancrivora*	Phatthalung, Thailand	FMNH 175923	–	B	This study
*F. raja*	*F. cancrivora*	Phatthalung, Thailand	FMNH 175924	–	B	This study
*F. raja*	*F. cancrivora*	Phatthalung, Thailand	FMNH 175925	–	B	This study
*F. raja*	*F. cancrivora*	Phatthalung, Thailand	FMNH 175926	–	B	This study
*F. raja*	*F. cancrivora*	Songkhla, Thailand	THNHM 04955	–	B	This study
*F. raja*	*F. cancrivora*	Songkhla, Thailand	THNHM 04956	–	B	This study
*F. raja*	*F. cancrivora*	Nong Chick, Pattani, Thailand	THNHM 15623	–	B	This study
*F. raja*	*F. cancrivora*	Su-Ngai Kolok, Narathiwat, Thailand	THNHM 19221	–	B	This study
*F. raja*	*F. cancrivora*	Tak Bai, Narathiwat, Thailand	THNHM 19771	–	B	This study
*F. raja*	*F. cancrivora*	Tak Bai, Narathiwat, Thailand	THNHM 19765	–	B	This study
*F. raja*	*F. cancrivora*	Tak Bai, Narathiwat, Thailand	THNHM 19766	–	B	This study
*F. raja*	*F. cancrivora*	Tak Bai, Narathiwat, Thailand	THNHM 19767	–	B	This study
*F. raja*	*F. cancrivora*	Tak Bai, Narathiwat, Thailand	THNHM 19768	–	B	This study
*F. raja*	*F. cancrivora*	Tak Bai, Narathiwat, Thailand	THNHM 19769	–	B	This study
*F. raja*	*F. cancrivora*	Tak Bai, Narathiwat, Thailand	THNHM 19770	–	B	This study
*F. raja*	*F. cancrivora*	Pak Phayun, Phatthalung, Thailand	THNHM 19852	–	B	This study
*F. raja*	*F. cancrivora*	Pak Phayun, Phatthalung, Thailand	THNHM 19853	–	B	This study
*F. raja*	*F. cancrivora*	Pak Phayun, Phatthalung, Thailand	THNHM 19854	–	B	This study
*F. raja*	*F. cancrivora*	Pak Phayun, Phatthalung, Thailand	THNHM 19855	–	B	This study
*F. raja*	*F. cancrivora*	Pak Phayun, Phatthalung, Thailand	THNHM 19857	–	B	This study
*F. raja*	*F. cancrivora*	Su-Ngai Kolok, Narathiwat, Thailand	THNHM 20754	–	B	This study
*F. raja*	*F. cancrivora*	Nong Chick, Pattani, Thailand	THNHM 21248	–	B	This study
*F. raja*	*F. cancrivora*	Pak Phanang, Nakhon Si Thammarat, Thailand	THNHM 25499	–	B	This study
*F. raja*	*F. cancrivora*	Khuan Khanun, Phatthalung, Thailand	ZMKU AM 01418	MN453520	A	This study
*F. raja*	*F. cancrivora*	Khuan Khanun, Phatthalung, Thailand	ZMKU AM 01423	MN453521	A, B	This study
*F. raja*	*F. cancrivora*	Khuan Khanun, Phatthalung, Thailand	ZMKU AM 01424	–	B	This study
*F. raja*	*F. cancrivora*	Khuan Khanun, Phatthalung, Thailand	ZMKU AM 01425	MN453522	A	This study
*F. raja*	*F. cancrivora*	Khuan Khanun, Phatthalung, Thailand	ZMKU AM 01426	MN453523	A, B	This study
*F. raja*	*F. cancrivora*	Khuan Khanun, Phatthalung, Thailand	ZMKU AM 01429	–	B	This study
*F. raja*	*F. cancrivora*	Khuan Khanun, Phatthalung, Thailand	ZMKU AM 01430	MN453524	A, B	This study
*F. raja*	*F. cancrivora*	Khuan Khanun, Phatthalung, Thailand	ZMKU AM 01432	–	B	This study
*F. raja*	*F. cancrivora*	Pak Phanang, Nakhon Si Thammarat, Thailand	ZMKU AM 01507	MN453525	A, B	This study
*F. raja*	*F. cancrivora*	Pak Phanang, Nakhon Si Thammarat, Thailand	ZMKU AM 01508	–	B	This study
*F. raja*	*F. cancrivora*	Pak Phanang, Nakhon Si Thammarat, Thailand	ZMKU AM 01509	MN453526	A, B	This study
*F. raja*	*F. cancrivora*	Pak Phanang, Nakhon Si Thammarat, Thailand	ZMKU AM 01510	–	B	This study
*F. raja*	*F. cancrivora*	Pak Phanang, Nakhon Si Thammarat, Thailand	ZMKU AM 01511	MN453527	A, B	This study
*F. raja*	*F. cancrivora*	Pak Phanang, Nakhon Si Thammarat, Thailand	ZMKU AM 01512	–	B	This study
*F. raja*	*F. cancrivora*	Pak Phanang, Nakhon Si Thammarat, Thailand	ZMKU AM 01513	–	B	This study
*Fejervarya* sp.	*Fejervarya* sp.	Pelabuhan ratu, Java, Indonesia	–	AB444693	A	Kurniawan et al. (2010)
*Fejervarya* sp.	*Fejervarya* sp.	Makassar, Sulawesi, Indonesia	–	AB570278	A	Kurniawan et al. (2014)
*Fejervarya* sp.	*Fejervarya* sp.	Makassar, Sulawesi, Indonesia	–	AB570288	A	Kurniawan et al. (2014)
*F. cancrivora*	*Fejervarya* sp.	Selatan, Sulawesi, Indonesia	–	EU979849	A	Che et al. (2009)
*F. iskandari*	*F. iskandari*	Malang, Java, Indonesia	–	AB570268	A	Kurniawan et al. (2014)
*F. limnocharis*	*F. limnocharis*	Java, Indonesia	–	AB277292	A	Kotaki et al. (2008)
*F. multistriata*	*F. multistriata*	Yunan, China	–	AB354237	A	Djong et al. (2011)
*F. vittigera*	*F. vittigera*	Quezon, Luzon Island, Philippines	–	AY313683	A	Evans et al. (2003)
*Euphlyctis cyanophlyctis*	*E. cyanophlyctis*	Mangalore, India	–	AB488901	A	Kotaki et al. (2010)
*Limnonectes jarujini*	*L. jarujini*	Surat Thani, Thailand	–	AB558951	A	Matsui et al. (2010)
*Occidozyga lima*	*O. lima*	Kuala Lumpur, Malaysia	–	AB488903	A	Kotaki et al. (2010)

### DNA extraction, amplification and sequencing

Total genomic DNA was extracted from liver or muscle tissue using the GF-1 Tissue DNA Extraction Kit (Vivantis Inc.). A 961–962 bp fragment of mitochondrial (mt) DNA that encodes part of the 16S rRNA gene was amplified by the polymerase chain reaction (PCR; 94 °C 45s, 58 °C 30s, 72 °C 1 min) for 35 cycles using the primer pairs L16SRanaIII (Stuart et al. 2006a) and 16Sbr3’ (Palumbi 1996). PCR products were purified using the NucleoSpin Gel and PCR Clean-up (MachereyNagel Inc.) and sequenced in both directions on an ABI 3730XL DNA analyzer by Bioneer Inc. (Daejeon, Korea) using Big Dye version 3 chemistry, the amplifying primers, and the internal primers H-16SRanaIII (Stuart et al. 2006a) and 16Sar-3’ (Palumbi 1996). DNA sequences were edited and aligned using Geneious v7.0.6 (Biomatter, Ltd.), and deposited in GenBank under accession numbers MN453492–MN453527 (Table 1).

### Phylogenetic analysis

Homologous sequences of *F. cancrivora* and *F. moodiei*, and the outgroup taxa *F. iskandari* Vieth, Kosuch, Ohler & Dubois, 2001, *F. limnocharis* (Gravenhorst, 1829), *F. multistriata* (Hallowell, 1861), *F. vittigera* (Wiegmann, 1834), *Euphlyctis cyanophlyctis* (Schneider, 1799), *Limnonectes jarujini* Matsui, Panha, Khonsue & Kuraishi, 2010, and *Occidozyga lima* (Gravenhorst, 1829) (following Islam et al. 2008; Kotaki et al. 2010; Kurniawan et al. 2010; Hasan et al. 2014), were downloaded from GenBank (Table 1). Downloaded sequences were trimmed to match the length of the 16S fragment obtained here and aligned to the newly-generated sequences using the MUSCLE plug-in as implemented in Geneious v 7.0.6. The best-fit nucleotide substitution model for the dataset was inferred to be GTR+I+G using the Akaike information criterion (AIC) as implemented in jModelTest v2.1.10 (Darriba et al. 2012). Phylogenetic analyses were performed using Bayesian inference with MrBayes 3.2.1 (Ronquist et al. 2012). Two independent runs, each with four Markov Chain Monte Carlo (MCMC) chains, were executed for 10 million generations using the default priors, trees were sampled every 1,000 generations, and the first 25% of trees were discarded as ‘burn-in.’ A 50% majority-rule consensus of the sampled trees was constructed to calculate the posterior probabilities of the tree nodes. Run parameters, stationarity and convergence were assessed using the program Tracer v.1.7 (Rambaut et al. 2018). Uncorrected pairwise sequence divergences (*p*-distances) were calculated in MEGA X (Kumar et al. 2018).

### Morphological study

Morphological analyses were performed on 108 sexually mature individuals (61 males, 47 females) of *F. cancrivora*, *F. moodiei*, and *F. raja* (Table 1; Appendix 2, 3). Importantly, these included the neotype (FMNH 256688) and topotypes of *F. cancrivora* from Java, Indonesia; the holotype of *F. moodiei* (CM 3724) from Luzon, Philippines; and topotypes of *F. raja* from Pattani, Thailand (Table 1). Sexual maturity was determined by presence of secondary characteristics, including nuptial pads or vocal sac folds in males, and convoluted oviducts or mature ova in females. Webbing formulae follow Savage and Heyer (1967).

Measurements were taken with digital Vernier calipers to the nearest 0.1 mm. Twenty-three morphological characters were measured following Djong et al. (2007) and Islam et al. (2008):

**EL** eye length, greatest diameter of the eye including upper eyelids,

**EN** distance from front of eye to nostril,

**FAL** forelimb length, from elbow to base of outer palmar tubercle,

**FOL** foot length, from base of inner metatarsal tubercle to tip of fourth toe,

**HAL** hand length, from base of outer palmar tubercle to tip of third finger,

**HL** head length, from back of mandible to tip of snout,

**HLL** hindlimb length,

**HW** head width, from left side back of mandible to right side back of mandible,

**IMTL** length of inner metatarsal tubercle,

**IN** internarial space, distance between the nostrils,

**IOD** interorbital distance,

**ITL** inner toe length,

**NS** nostril-snout length, distance from nostril to tip of snout,

**NTL** nostril-tympanum length, distance between nostril and front of tympanum,

**SL** snout length, distance from front of eye to tip of snout,

**STL** snout-tympanum length, tip of snout to front of tympanum,

**SVL** snout-vent length,

**TD** tympanum diameter, maximum diameter,

**TEL** tympanum-eye length, distance between end of eye to front of tympanum,

**TFOL** length of tarsus and foot, from base of tarsus to tip of fourth toe,

**THIGHL** thigh length,

**TL** tibia length,

**UEW** maximum width of upper eyelids,

**1FL** first finger length.

Qualitative characters were taken on the presence and condition of the vomerine ridge, skin on dorsum, coloration and pattern on dorsum, vocal sac pigmentation, fejervaryan lines (conspicuous ventrolateral lines on the ventral side of the body), tubercles on forelimbs and hindlimbs, dermal fringe on fingers II and III, inner tarsal ridge, dermal flap on outer side of Toe V, and foot webbing.

To correct for body size, each mensural character was divided by SVL to a ratio (r) and then converted to a percentage. Specimens were assigned to group (= species) based on their mtDNA assignment (below). Principal component analysis (PCA) was performed separately by sex using FactoMineR and factoextra R package (Lê et al. 2008; Husson et al. 2017) in the R programs v.3.4.3 (R Core Team 2017) to assess morphometric differences between groups. All variables were tested for normality using Shapiro-Wilk’s test. Statistical differences between species were tested by *t*-test for parametric data and Mann-Whitney U test for non-parametric data at a significance level of 95%.

## Results

### Phylogenetic analyses

**Figure 2.**
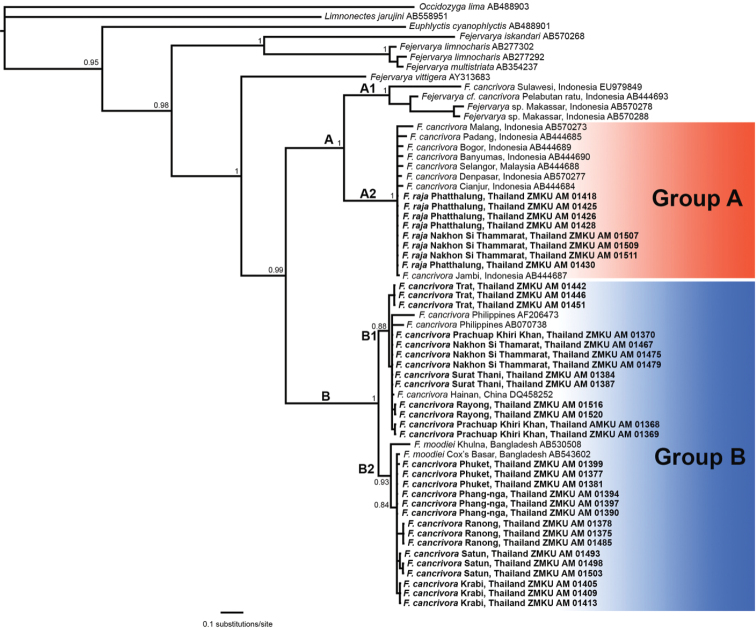
Bayesian consensus phylogram of the mitochondrial16S rRNA gene of *Fejervarya cancrivora* and the closely related species, *F. moodiei* and *F. raja*. Numbers at nodes represent Bayesian posterior probability support values. Clade and subclade names are presented next to branches and group names are presented to the right of terminal taxa.

The aligned dataset contained 61 individuals and 981 characters. The standard deviation of split frequencies was 0.003331 among the two Bayesian runs, and the Estimated Sample Sizes (ESS) of parameters were ≥ 200. The Bayesian analysis recovered the *F. cancrivora* complex as monophyletic with strong support, and to contain two major clades referred to as Clades A and B (Fig. 2). Clade A contained subclade A1 consisting of F. cf. cancrivora from Indonesia (Pelabuhan Ratu and Sulawesi) and subclade A2 consisting of *F. cancrivora* from Indonesia (Sumatra, Java, Bali) and Malaysia (Selangor), as well as *F. raja* from Thailand (Phatthalung, Nakhon Si Thammarat). Clade B contained subclade B1 consisting of *F. cancrivora* from Thailand (Trat, Nakhon Si Thammarat, Surat Thani, Prachuap Khiri Khan, Rayong), Philippines and China, and subclade B2 consisting of *F. cancrivora* from Thailand (Phuket, Phang-nga, Ranong, Satun, Krabi) and *F. moodiei* from Bangladesh (Cox’s Barza, Khulna).

**Figure 3.**
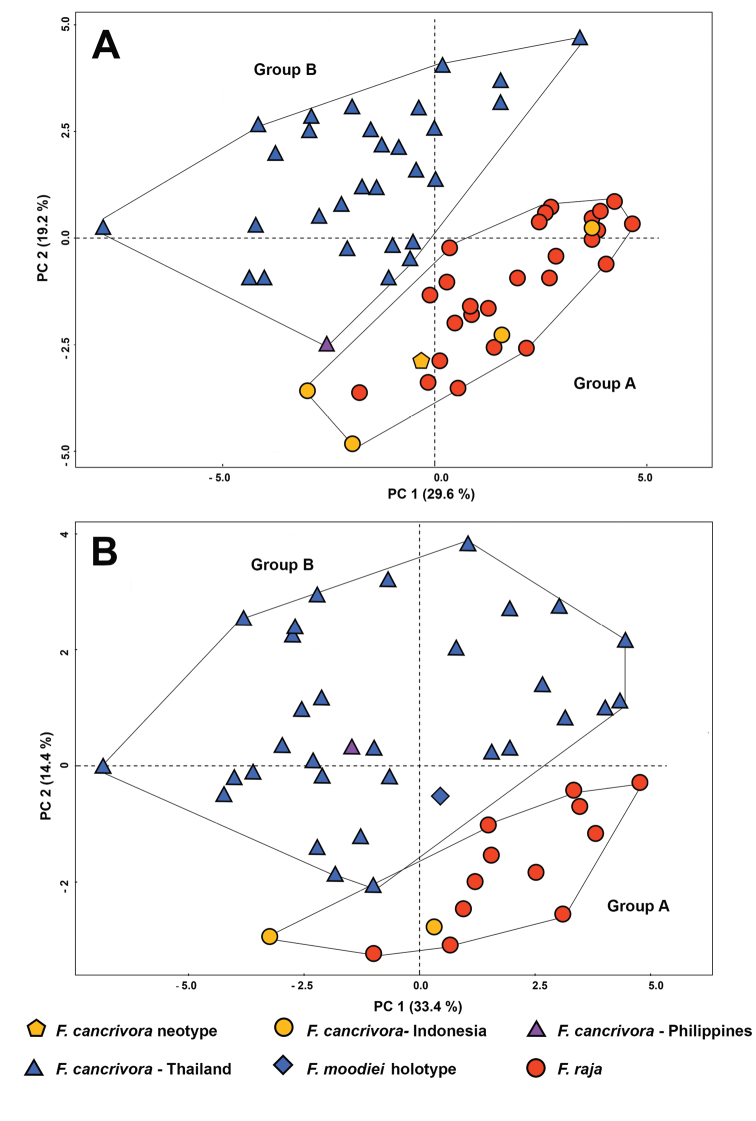
Principal component analysis of morphological measurements from males (**A**) and females (**B**) of *Fejervarya cancrivora*, *F. moodiei*, and *F. raja*.

Uncorrected pairwise sequence divergences (*p*-distances) were relatively low within subclades, with subclade A1 ranging from 0.6–6.0% (mean 3.6%), subclade A2 ranging from 0.0–1.4% (mean 0.3%), and subclades B1 and B2 each ranging from 0.0–1.6% (means 0.4%; Table 2). In contrast, genetic distances were relatively high between subclades (6.5–10.5%) except for subclades B1 and B2 (mean 1.7%; Table 2). As such, we refer to subclade A2 as “*F. cancrivora* Group A,” and to the merged subclades B1 and B2 as “*F. cancrivora* Group B” (Fig. 3).

**Table 2.** Uncorrected pairwise sequence divergences (*p*-distances) in the mitochondrial 16S rRNA gene of *Fejervarya cancrivora* and related species. Mitochondrial subclades A1, A2, B1, and B2 are defined in the text.

	*** iskandari ***	*** multistriata ***	*** limnocharis ***	*** vittigera ***	***cancrivora* B2**	***cancrivora* B1**	***cancrivora* A2**	**sp. A1**
*** iskandari ***	–							
*** multistriata ***	(12.8)	–						
	12.8							
*** limnocharis ***	(12.1–12.7)	(0.2–0.4)	(0.9)					
	12.4	0.3	0.9					
*** vittigera ***	(16.2)	(12.2)	(11.7–13.5)	–				
	16.2	12.2	12.6					
***cancrivora* B2**	(17.8–18.2)	(13.7–13.9)	(13.4–15.0)	(11.4–12.3)	(0.0–1.6)			
	18.0	13.8	13.9	11.5	0.4			
***cancrivora* B1**	(14.3–18.6)	(13.9–14.2)	(13.4–14.8)	(9.5–12.9)	(0.9–3.4)	(0.0–1.6)		
	17.4	14.1	14.0	11.7	1.7	0.4		
***cancrivora* A2**	(12.5–17.1)	(13.9–14.3)	(13.4–15.1)	(10.7–12.8)	(8.8–10.7)	(8.3–11.1)	(0.0–1.4)	
	15.5	13.7	14.1	11.9	9.7	9.3	0.3	
**sp. A1**	(10.9–12.5)	(12.8–13.7)	(12.3–13.5)	(9.5–10.2)	(9.8–11.0)	(8.9–11.0)	(4.5–7.9)	(0.6–6.0)
	11.7	13.4	13.2	9.8	10.5	9.3	6.5	3.6

## Morphological analyses

PCA analysis of males revealed morphometric differences between *F. cancrivora* Group A and *F. cancrivora* Group B, with no overlap on a plot of the first two axes (Fig. 3A). The first three principal components (PC) of males with Eigenvalues > 1.0 accounted for a cumulative 61.2% of the total variance (29.6% by PC1, 19.2% by PC2 and 12.4% by PC3; Table 3). PC1 was heavily and positively loaded on rTL, rHW, rFOL, rTHIGHL, rTFOL, and rSL. PC2 was heavily and positively loaded on rEL, rTD, rNTL, and negatively on SVL, suggesting a strong negative correlation between these characters. PC3 was heavily and positively loaded on r1FL. These results indicated that PC1 and PC2 were strongly influenced by body size. Males of *F. cancrivora* Group A had larger SVL, rTL, rHW, rFOL, rTHIGHL, rTFOL, and rSL, but smaller rEL, rTD, and rNTL than males of Group B based on scores of the first two axes (Fig. 3A).

PCA analysis of females revealed morphometric differences between *F. cancrivora* Group A and *F. cancrivora* Group B, with only slight overlap on a plot of the first two axes (Fig. 3B). The first three PCs of females with Eigenvalues > 1.0 accounted for a cumulative 35.3% of the total variance (35.3% by PC1, 14.5% by PC2 and 9.3% by PC3; Table 3). PC1 was heavily and positively loaded on rSL, rFOL, rTFOL, rSTL, rTL, rITL, rEN, and rHAL, indicating that it was strongly influenced by body size. PC2 was heavily and positively loaded on rEL and negatively on SVL, implying a strong negative correlation between these characters. PC3 was moderately and positively loaded on rUEW and negatively on r1FL. Females of *F. cancrivora* Group A had larger SVL rSL, rFOL, rTFOL, rSTL, rTL, rITL, rEN, and rHAL, but smaller rEL than females of Group B based on scores of the first two axes (Fig. 3B).

**Table 3.** Factor loading on the first three principal components of 23 morphological characters for male and female *Fejervarya cancrivora*, *F. moodiei*, and *F. raja*.

Character	Males			Females		
	PC 1	PC 2	PC 3	PC 1	PC 2	PC 3
SVL	0.395	-0.829	-0.211	0.136	-0.921	-0.008
rHL	0.614	0.462	-0.306	0.628	0.230	0.254
rHW	0.795	-0.093	-0.262	0.660	-0.445	0.297
rSTL	0.623	0.610	-0.222	0.762	0.201	0.353
rNS	0.272	0.574	-0.157	0.640	0.252	0.213
rSL	0.725	0.300	-0.343	0.829	-0.119	0.162
rNTL	0.511	0.703	-0.155	0.654	0.165	0.310
rEN	0.601	0.108	-0.347	0.715	0.016	0.240
rTEL	0.376	-0.236	-0.255	0.329	-0.589	-0.243
rTD	-0.211	0.744	-0.213	0.199	0.570	0.149
rIN	0.166	-0.041	-0.319	0.562	-0.185	0.136
rEL	-0.279	0.767	0.100	0.064	0.820	0.128
rIOD	-0.278	0.659	0.431	0.055	0.628	-0.356
rUEW	0.132	0.176	-0.556	0.104	0.286	0.575
rHAL	0.549	0.358	0.487	0.701	0.285	-0.447
rFAL	0.157	0.408	0.538	0.422	0.074	-0.325
rTHIGHL	0.768	-0.128	-0.138	0.675	-0.136	0.146
rTL	0.815	-0.384	-0.106	0.760	-0.281	0.208
rFOL	0.775	-0.011	0.421	0.800	0.055	-0.250
rTFOL	0.766	-0.249	0.382	0.766	-0.164	-0.135
r1FL	0.481	-0.163	0.664	0.657	0.110	-0.578
rIMTL	0.436	0.034	0.252	0.478	0.089	-0.383
rITL	0.674	-0.029	0.466	0.758	0.064	-0.373
Elegenvalue	6.807	4.420	2.860	8.112	3.337	2.146
Percentage of variance	29.595	19.218	12.435	35.268	14.508	9.331
Cumulative proportion	29.595	48.813	61.248	35.268	49.776	59.107

Summary statistics of morphological characters of adult males and females are shown in Table 4. The *t*-tests and Mann-Whitney U tests found significant differences (*p* < 0.05–0.0001). Males of *F. cancrivora* Groups A and B were significantly different in most morphometric characters (*t*-tests and Mann-Whitney U tests, *p* < 0.05–0.0001), including body size (SVL), head (rHW), snout (rSL), eye (rEL, rEN, rTEL, rIOD), tympanum (rTD), and hindlimbs (rTHIGHL, rTL, rFOL, rTFOL; Table 4). Females of *F. cancrivora* Groups A and B were also significantly different (*p* < 0.05–0.0001) in most morphometric characters, including body size (SVL), head (rHW), snout (rSL), nostril (rIN), eye (rEN, rTEL, rEL, rIOD), and hindlimb (rTL, rTFOL; Table 4). Comparisons of morphometric measurements of adult males and females are given in Appendix 2, 3.

**Table 4.** Comparisons of body sizes of *Fejervarya cancrivora* and *F. moodiei*. Data are given as mean and standard deviation, followed by range in parentheses. Key: ^a^ tested by Mann-Whitney U test, ^*^ significance level at *p* < 0.05.

Characters	Males				Females			
	*F. cancrivora*	*F. moodiei*	*t*-test	*p*	*F. cancrivora*	*F. moodiei*	*t*-test	*p*
	*n* = 31	*n* = 30			*n* = 14	*n* = 33		
SVL	71.3 ± 5.6	51.4 ± 5.4	-13.826	< 0.0001*	94.2 ± 6.5	69.0 ± 10.1	0^a^	< 0.0001*
	(60.2–79.8)	(42.7–62.7)			(85.1–107.1)	(50.0–81.8)		
rHL	40.8 ± 1.8	39.9 ± 1.7	340^a^	0.0692	39.6 ± 2.4	39.4 ± 1.7	201.5^a^	0.4953
	(36.7– 43.5)	(37.2– 44.5)			(35.2–42.9)	(35.9–42.2)		
rHW	37.1 ± 1.8	34.6 ± 1.1	-6.553	< 0.0001*	38.2 ± 1.6	35.6 ± 1.9	65.5^a^	0.0001*
	(32.5–40.6)	(32.4–37.1)			(35.0–41.0)	(32.5–38.7)		
rSTL	30.3 ± 1.2	30.1 ± 1.0	414.500^a^	0.4609	29.9 ± 1.1	29.3 ± 1.0	-1.867	0.0684
	(27.5–32.1)	(28.5–32.0)			(27.9–31.6)	(27.6–31.3)		
rNS	7.2 ± 0.6	7.4 ± 0.5	1.473	0.1460	7.3 ± 0.8	7.1 ± 0.6	177^a^	0.2115
	(5.9–8.5)	(6.1– 8.8)			(5.4–8.2)	(6.1–8.5)		
rSL	17.0 ± 1.0	16.4 ± 0.7	262.500^a^	0.0033*	17.2 ± 0.9	16.1 ± 0.8	88^a^	0.0008*
	(14.7–18.2)	(15.2–17.9)			(15.2–18.3)	(14.6–17.9)		
rNTL	23.28 ± 0.9	23.4 ± 1.1	0.402	0.6893	23.0 ± 0.7	22.7 ± 0.9	-0.783	0.4375
	(21.6–25.3)	(21.5–25.8)			(21.8 – 24.0)	(21.2–24.4)		
rEN	9.5 ± 0.5	8.8 ± 0.8	-3.759	0.0004*	9.5 ± 0.4	8.8 ± 0.7	95^a^	0.0015*
	(8.4–10.7)	(7.4 – 11.3)			(8.5–10.1)	(7.5–10.1)		
rTEL	3.83 ± 0.64	3.23 ± 0.62	-3.840	0.0003*	4.83 ± 0.64	4.28 ± 0.83	-2.295	0.0265*
	(2.73 – 5.21)	(2.40 – 5.05)			(4.06 – 6.32)	(2.76 – 5.98)		
rTD	7.2 ± 0.5	7.9 ± 0.6	4.840	< 0.0001*	6.9 ± 0.4	7.1 ± 0.6	1.020	0.3131
	(6.4–8.0)	(6.8– 9.3)			(6.2–7.9)	(5.7– 8.0)		
rIN	4.9 ± 0.4	4.9 ± 0.6	371^a^	0.1750	4.9 ± 0.4	4.6 ± 0.4	-2.270	0.0281*
	(4.17 – 5.62)	(3.8–6.2)			(4.3–6.0)	(3.8–5.5)		
rEL	9.8 ± 0.7	11.5 ± 1.1	7.026	< 0.0001*	8.9 ± 0.9	10.1 ± 1.0	3.850	0.0004*
	(8.1–11.2)	(9.2–13.4)			(7.5–10.3)	(8.3–12.4)		
rIOD	4.8 ± 0.6	6.2 ± 0.7	7.902	< 0.0001*	5.0 ± 0.4	5.6 ± 0.7	3.158	0.0028*
	(3.7 – 5.9)	(4.6– 8.2)			(4.1–5.51)	(4.1–7.5)		
rUEW	8.3 ± 0.7	8.2 ± 0.6	-0.270	0.7882	8.0 ± 0.8	7.9 ± 0.7	-0.140	0.8895
	(6.8–9.6)	(7.1–9.5)			(6.4–9.4)	(6.3–9.2)		
rHAL	24.6 ± 0.9	24.7 ± 1.1	0.081	0.9359	23.9 ± 1.2	24.0 ± 1.5	218^a^	0.7692
	(23.2–26.3)	(21.3–26.8)			(21.2–25.6)	(21.3–27.45)		
rFAL	19.3 ± 0.9	19.8 ± 1.2	1.609	0.1130	18.7 ± 0.8	18.8 ± 1.3	0.084	0.9335
	(17.6–21.4)	(17.8– 22.5)			(17.4–20.0)	(16.7–21.4)		
rTHIGHL	47.8 ± 1.93	45.5 ± 1.9	178.5^a^	< 0.0001*	46.0 ± 2.4	43.6 ± 2.1	92^a^	0.0012
	(42.1–51.1)	(42.6–49.3)			(40.0–48.5)	(39.9–47.6)		
rTL	52.0 ± 1.4	47.6 ± 2.2	53^a^	< 0.0001*	50.8 ± 3.0	46.4 ± 2.3	62.5^a^	< 0.0001*
	(48.7–55.6)	(41.0–53.0)			(42.7–54.13)	(43.4 – 50.7)		
rFOL	54.1 ± 2.2	51.8 ± 3.0	261.5^a^	0.0027*	52.1 ± 1.5	50.8 ± 3.1	174^a^	0.1842
	(49.8–57.8)	(43.4–58.3)			(50.0–55.0)	(44.1–55.2)		
rTFOL	79.6 ± 3.3	75.1 ± 4.0	162^a^	< 0.0001*	77.6 ± 4.0	72.9 ± 4.6	109.5^a^	0.004616*
	(73.6–86.5)	(63.6– 81.6)			(71.4–87.0)	(66.3–81.2)		
r1FL	18.9 ± 1.3	18.2 ± 1.3	-1.967	0.0539	19.0 ± 1.1	18.9 ± 1.1	-0.173	0.8637
	(17.2–21.2)	(16.2–20.8)			(16.6–20.6)	(17.0–21.0)		
rIMTL	6.0 ± 0.6	5.8 ± 0.6	342.5^a^	0.0773	5.9 ± 0.5	6.0 ± 0.5	252^a^	0.6317
	(4.0–6.9)	(4.2–7.0)			(4.7–6.5)	(4.8–6.7)		
rITL	18.6 ± 1.1	17.9 ± 1.8	327.5^a^	0.0469*	18.4 ± 1.1	18.1 ± 1.4	-0.901	0.3724
	(15.1–20.1)	(14.8–21.8)			(15.9–19.8)	(15.2–20.7)		
HL/HW	1.1 ± 0.0	1.2 ± 0.0	4.913	< 0.0001*	1.0 ± 0.0	1.1 ± 0.1	4.462	< 0.0001*
	(1.0–1.2)	(1.1–1.2)			(1.0–1.1)	(1.0–1.2)		
IOD/HW	0.1 ± 0.0	0.2 ± 0.0	10.343	< 0.0001*	0.1 ± 0.0	0.2 ± 0.0	4.619	< 0.0001*
	(0.1–0.2)	(0.1–0.2)			(0.1– 0.2)	(0.1– 0.2)		
SL/HL	0.4 ± 0.0	0.4 ± 0.0	-1.448	0.1529	0.43 ± 0.0	0.4 ± 0.0	-4.426	< 0.0001*
	(0.4–0.5)	(0.4–0.5)			(0.4–0.5)	(0.4–0.5)		
EL/HL	0.2 ± 0.0	0.3 ± 0.0	8.662	< 0.0001*	0.2 ± 0.0	0.3 ± 0.0	375^a^	0.0008*
	(0.2–0.3)	(0.2–0.3)			(0.2–0.3)	(0.2–0.3)		
NS/EN	0.8 ± 0.1	0.8 ± 0.1	4.505	< 0.0001*	0.8 ± 0.1	0.8 ± 0.1	2.278	0.0275*
	(0.6–0.9)	(0.7–1.0)			(0.6–0.9)	(0.7–0.9)		
EL/SL	0.6 ± 0.0	0.7 ± 0.1	8.775	< 0.0001*	0.5 ± 0.1	0.6 ± 0.1	5.664	< 0.0001*
	(0.5–0.7)	(0.6–0.8)			(0.5–0.6)	(0.5–0.8)		
EL/EN	1.0 ± 0.1	1.3 ± 0.2	9.196	< 0.0001*	0.9 ± 0.1	1.1 ± 0.1	6.303	< 0.0001*
	(0.9–1.2)	(1.0–1.6)			(0.8–1.1)	(1.0–1.4)		
IN/IOD	1.0 ± 0.2	0.8 ± 0.1	-6.372	< 0.0001*	1.0 ± 0.1	0.8 ± 0.1	-3.839	0.0004*
	(0.8–1.5)	(0.5–1.2)			(0.8–1.1)	(0.6–1.3)		
TD/EL	0.7 ± 0.1	0.7 ± 0.1	-3.201	0.0022*	0.8 ± 0.1	0.7 ± 0.1	-2.754	0.0085*
	(0.6–0.9)	(0.6–0.8)			(0.6–0.9)	(0.6–0.9)		
TEL/EL	0.4 ± 0.1	0.3 ± 0.1	-6.585	< 0.0001*	0.6 ± 0.1	0.4 ± 0.1	108^a^	0.0044*
	(0.3–0.6)	(0.2–0.4)			(0.4–0.8)	(0.2–0.6)		
FAL/HAL	0.8 ± 0.0	0.8 ± 0.1	1.385	0.1712	0.8 ± 0.0	0.8 ± 0.1	0.031	0.9754
	(0.7–0.9)	(0.7–0.9)			(0.7–0.8)	(0.7– 0.9)		
THIGHL/TL	0.9 ± 0.0	1.0 ± 0.0	741^a^	< 0.0001*	0.9 ± 0.1	0.9 ± 0.0	2.574	0.0134*
	(0.9–1.0)	(0.9–1.1)			(0.8–1.0)	(0.9–1.0)		
FOL/TL	1.0 ± 0.0	1.1 ± 0.1	755^a^	< 0.0001*	1. ± 0.1	1.01 ± 0.1	405^a^	< 0.0001*
	(1.0–1.1)	(0.9–1.2)			(1.0–1.2)	(1.0–1.2)		
IMTL/TL	0.1 ± 0.0	0.1 ± 0.0	615.5^a^	0.0296	0.1 ± 0.0	0.1 ± 0.0	3.342	0.0017*
	(0.1–0.1)	(0.1–0.2)			(0.1–0.1)	(0.1–0.2)		

### Species accounts

The genetic and morphometric data provide congruent, independent lines of evidence to support the hypothesis that *F. cancrivora* Groups A and B represent two separate species. Specifically, Group A consists of a composite of *F. cancrivora* from Indonesia and Malaysia, and *F. raja* from Thailand (Smith 1930; Taylor 1962; Chan-ard 2003; Chuaynkern and Chuaynkern 2012), while Group B consists of a composite of *F. cancrivora* and *F. moodiei* from Thailand, Philippines, China, and Bangladesh (Smith 1930; Taylor 1962; Chan-ard 2003; Kurniawan et al. 2010, 2011; Chuaynkern and Chuaynkern 2012; Table 1). We propose that Group A be referred to as *F. cancrivora* sensu stricto, with *F. raja* treated as a junior synonym of *F. cancrivora*. We propose that Group B be referred to as *F. moodiei*, with specimens of *F.* “*cancrivora*” in this clade reallocated to that species. The two species, *F. cancrivora* (Group A) and *F. moodiei* (Group B), can be recognized as follows.

#### Fejervarya cancrivora

Taxon classificationAnimaliaAnuraDicroglossidae

(Gravenhorst, 1829)

4CCD7164-33F4-53C5-82EB-B46F3D3DA62B

Rana cancrivora Gravenhorst, 1829: 41; Dubois and Ohler 2000: 30; Sumida et al. 2002: 294
Rana cancrivora raja Smith, 1930: 96
Rana raja Taylor, 1962: 373; Stuart et al. 2006: 19
Fejervarya cancrivora : Dubois & Ohler, 2000: 35; Kurniawan et al. 2014: 1
Fejervarya cancrivora : Large type Kurniawan et al. 2010: 222; Kurniawan et al. 2011: 12
Fejervarya raja : Chan-ard 2003: 110; Chuaynkern and Chuaynkern 2012: 169

##### Diagnosis.

*Fejervarya cancrivora* can be characterized by the following combination of characters: (1) large size, SVL 60.2–79.8 mm in males, 85.1–107.1 mm in females (Table 4; Appendix 2, 3); (2) head length slightly greater than head width; (3) skin on dorsum and flank with spinules and glandular warts, with irregular skin folds not arranged in series; (4) relative finger lengths II < IV < I < III; (5) dermal fringe on Finger II and III; (6) prepollax indistinct; (7) palmar tubercles indistinct; (8) foot moderately webbed with webbing formula I1–11/2II1–2III1–2IV2–1V; (9) dermal flap on postaxial side of Toe V; (10) Fejervaryan lines absent; (11) inner metatarsal tubercles prominent; (12) inner tarsal ridge prominent on distal half to two-thirds of tarsus, and (13) vocal sacs in adult males with wrinkled skin covered by triangular, very dark brown blotches on each side of throat.

##### Description of neotype.

**Figure 4.**
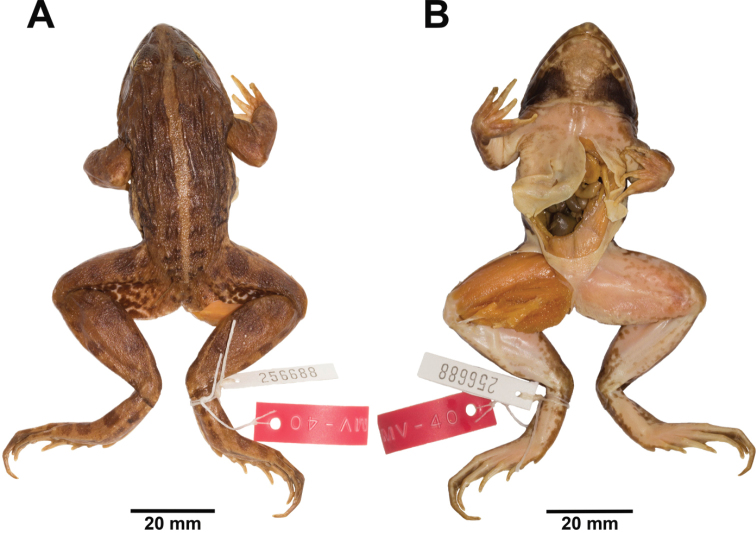
Adult male neotype of *Fejervarya cancrivora* (FMNH 256688) in preservative in **A** dorsal and **B** ventral views.

Dubios and Ohler (2000) designated and described the neotype adult male, FMNH 256688, from Java, Indonesia (Fig. 4A–B; Table 1). We supplement their description of the neotype, as follows: rather large size, body rather slender; head narrow, slightly longer than wide; snout oval in dorsal view, round in lateral view, projecting beyond lower jaw; nostril dorsolateral, pointed oval, with small lateral flap, closer to tip of snout than eye; canthus indistinct, rounded; loreal region concave and obtuse; eye diameter about 60% snout length; interorbital space flat, less than width of upper eyelid and internarial distance; pineal body visible; tympanum distinct, rounded [oval according to Dubois and Ohler (2000)], about 90% of eye diameter, not depressed relative to skin of temporal region, tympanic rim weakly elevated relative to tympanum, dorsoposterior margin obscured by supratympanic fold; two vomerine ridges bearing a few small teeth between choanae, obliquely oriented at an angle of 45° to body axis, closer to choanae than to each other; tongue large, cordate, emarginate [based on Ohler and Dubois (2000), not examined by us]; distinct supratympanic fold extending from eye to axilla, not obscuring dorsoposterior margin of tympanum.

Forelimbs short, rather stout [rather thin according to Dubois and Ohler (2000)], slightly longer than hand; fingers rather long, thin; tip of fingers slightly rounded and swollen [pointed according to Dubois and Ohler (2000)], but not expanded into discs; relative length of fingers II < IV < I < III; fingers II and III with dermal fringe; webbing on fingers absent; subarticular tubercles prominent, rounded; supernumerary tubercles absent; prepollex indistinct, oval; palmar tubercles indistinct.

**Figure 5.**
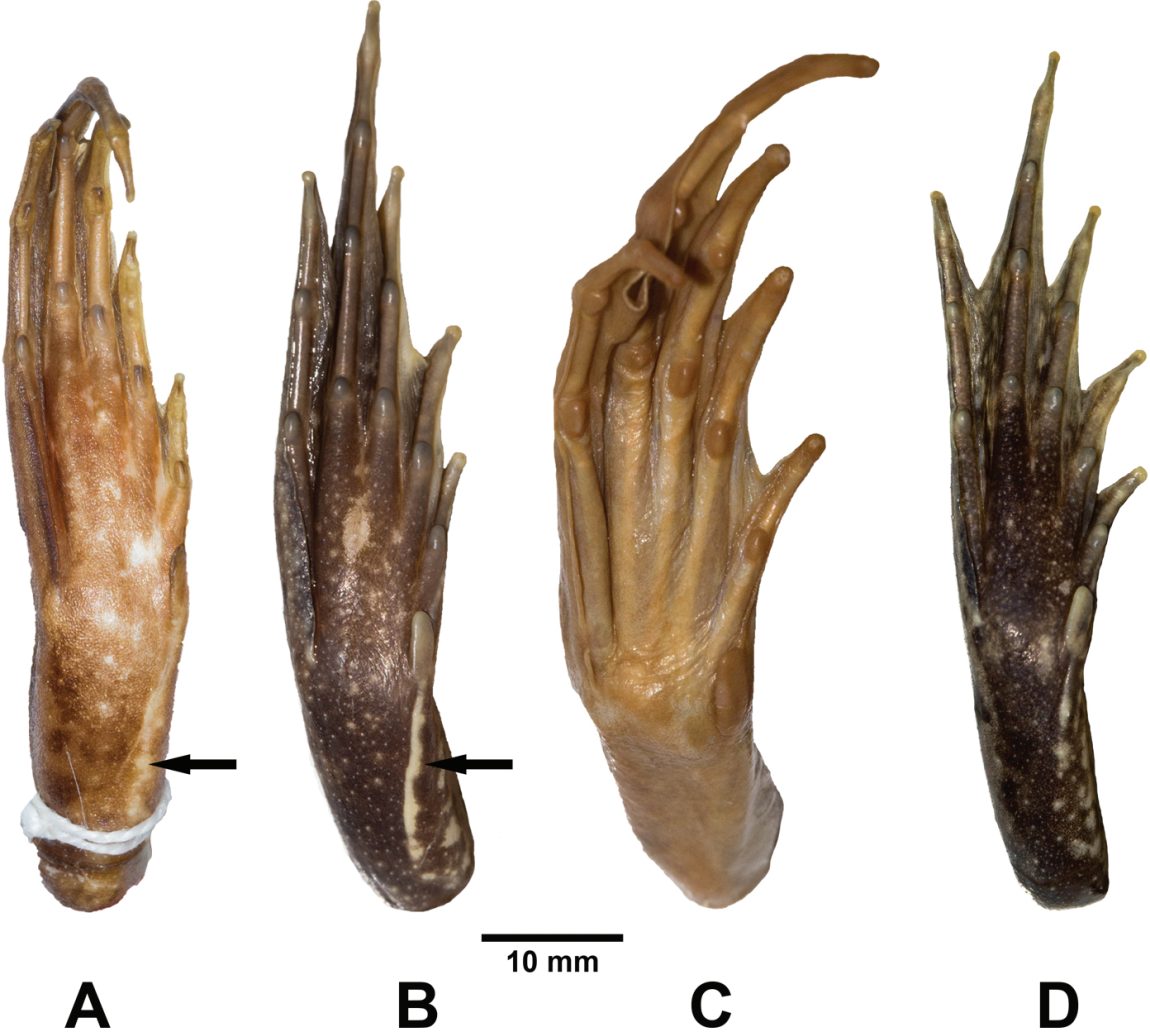
Plantar and metatarsal views of **A** adult male neotype of *Fejervarya cancrivora* (FMNH 256688) **B** adult male *F. cancrivora* (ZMKU AM 01426) from Khuan Khanun District, Phatthalung Province, Thailand **C** adult female holotype of *F. moodiei* holotype (CM 3724), and **D** adult male *F. moodiei* (ZMKU AM 10390) from Mueang Phang-nga District, Phang-nga Province, Thailand. The inner metatarsal ridge on the tarsus of *F. cancrivora* is indicated with an arrow.

Hindlimbs moderately short, robust; tibia longer than thigh, but shorter than distance from base of inner metatarsal tubercle to tip of Toe IV; toes long, thin; tips of toes rounded [pointed according to Dubois and Ohler (2000)], not expanded into discs; relative length of toes I < II < V < III < IV; webbing moderate, deeply excised between toes, formula I1–11/2 II1–1III1–2IV2–1V, Toe I webbed to base of distal phalanx; preaxial side of Toe II webbed to point between distal subarticular tubercle and distal phalanx, continuing as narrow fringe to base of distal phalanx; postaxial side of Toe II webbed to base of distal phalanx; preaxial side of Toe III webbed to distal subarticular tubercle, continuing as narrow fringe to base of distal phalanx, postaxial side of Toe III webbed to base of distal phalanx; preaxial side of Toe IV wedded to distal subarticular tubercle, continuing as narrow fringe to base of distal phalanx, postaxial side of Toe IV webbed to distal subarticular tubercle, continuing as narrow fringe to base of distal phalanx, Toe V webbed to base of distal phalanx; dermal flap well developed, extending along postaxial side of Toe V from level of inner metatarsal tubercles to distal phalanx; subarticular tubercles prominent; inner metatarsal tubercle prominent, oval, less than length of Toe I; distinct dermal ridge extending along inner metatarsal tubercle to distal phalanx of Toe I; distinct inner tarsal ridge on distal two-third of tarsus (Fig. 5A); outer metatarsal tubercles absent; supernumerary tubercles absent; tarsal tubercle absent.

Skin on snout and interorbital region shagreen; skin on eyelid with glandular warts and spinules; skin on dorsum with irregular skin folds, with intervening glandular warts and spinules; dorsolateral fold extending posteriorly to two-thirds length of dorsum; skin on side of head with small spinules; skin on flank with glandular warts; skin on cloacal region with dense glandular warts; skin on forelimbs, thigh, tibia and tarsus with glandular warts and spinules; skin on ventral surfaces smooth, except dense, fine spinules on chin. Nuptial pad with small translucent spinules on dorsal and medial surface of Finger I from base of distal phalanx to slightly over the base of prepollax; vocal sac present on both sides of throat, with wrinkled skin covered by triangular dark brown blotches. Fejervaryan lines absent.

##### Coloration of neotype in preservative.

Dorsum and side of head medium brown with indistinct dark brown markings; dark brown band between outer margins of upper eyelids; tympanum brown with inferior half more translucent, lighter in coloration than head; flank creamy white with dark brown marbling; three wide dark brown vertical spots on upper lips; wide light brown mid-dorsal stripe continuous from tip of snout to vent; dorsal surfaces of forelimbs, thigh, tibia, and foot brown with dark brown transverse spots; posterior surface of thighs with irregular pattern of dark brown marbling on white background; chin mottled dark brown, throat with triangular dark brown blotches on each side; chest, belly and ventral surfaces of hindlimbs creamy white with indistinct dark brown mottling; ventral surfaces of forelimbs creamy white; ventral surfaces of hand and foot brown; lower lip creamy white with dark brown spots.

##### Coloration of referred Thai specimen in life.

**Figure 6.**
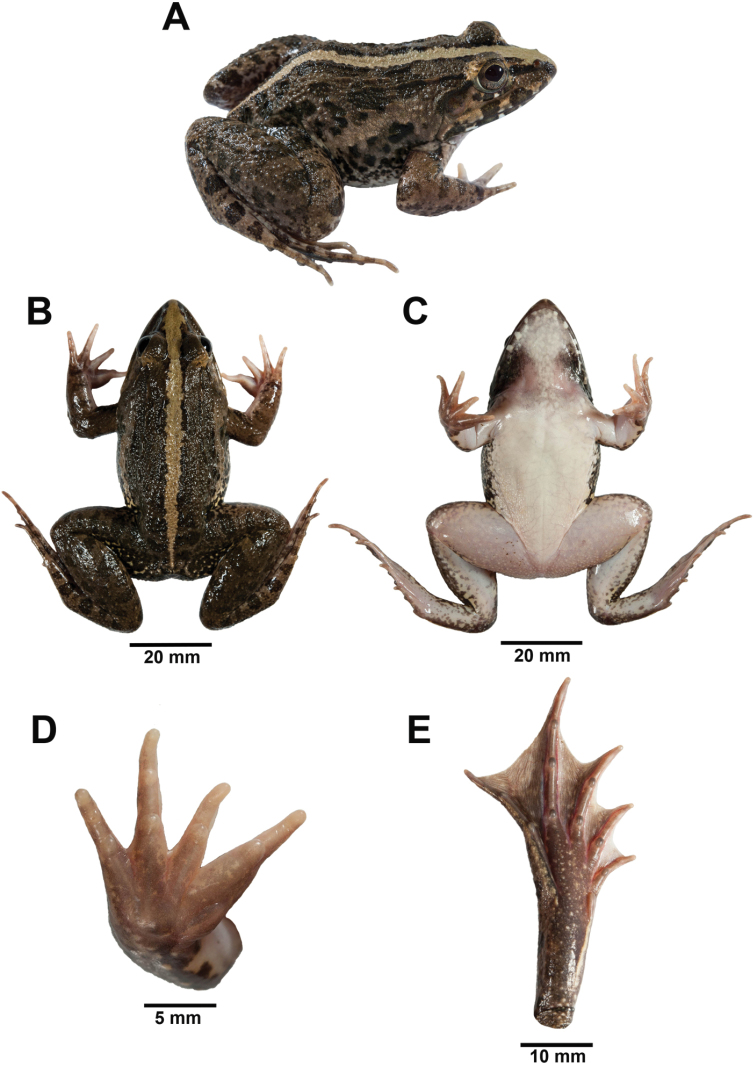
Adult male *Fejervarya cancrivora* (ZMKU AM 01426) from Khuan Khanun District, Phatthalung Province, Thailand (SVL = 66. 9 mm) immediately prior to preservation in **A** right lateral **B** dorsal **C** ventral **D** right palmar, and **E** right plantar views. Photographs by Attapol Rujirawan.

Adult male ZMKU AM 01426 (Fig. 6A–E) from Khuan Khanun District, Phatthalung Province, Thailand. SVL 60.3 mm. Dorsum dark brown with indistinct darker markings, side of head lighter brown; dark brown band between outer margins of upper eyelids; lower half of tympanum with brown blotches; dark brown streak on canthus rostralis from tip of snout to eye; dark brown streak from eye along supratympanic fold to posterior rim of tympanum; flank creamy white with dark brown marbling; three wide dark brown spots on upper lips; a wide beige mid-dorsal stripe continuous from tip of snout to vent; dorsal surfaces of forelimb, thigh, tibia, and foot dark brown with darker transverse spots; posterior part of thigh with irregular pattern of dark brown marbling on light brown background; chin and chest creamy white with dark brown mottling; throat with triangular dark brown blotches on each side; ventral surfaces of forelimbs and belly creamy white; ventral surfaces of hindlimbs creamy white with dark brown mottling; ventral surfaces of hand and foot brown; lower lip creamy white with dark brown spots.

##### Variations.

Females are distinctly larger in size (Table 4; Appendix 3), lack nuptial pads and vocal sacs, and have fewer spinules and glandular warts on dorsum and flanks than males. Two male specimens (ZMKU AM 01511 from Nakhon Si Thammarat Province, Thailand and CNHM 131100 from Java, Indonesia) have nuptial pads extending to the base of prepollax. Most male specimens have dense fine spinules over the entire surface of the chest, belly, and ventrolateral surface.

The examined male and female specimens closely resemble the neotype in morphology, with most observed variation pertaining to coloration. Dorsal coloration in preservative varied from medium to very dark brown with darker markings. Markings or spots on dorsum, and transverse spots on dorsal surface of forelimbs and hindlimbs fainter than neotype in some individuals. Flank pale brown with dark brown marbling in some individuals. Ventral coloration pale brown in some individuals, with dark mottling on chin and chest. Ventral surface of hand pale brown or creamy white in some individuals. Dorsal vertebral stripe present (*n* = 18, 41%) or absent (*n* = 26, 59%). Two specimens from Nakhon Si Thammarat Province, Thailand (ZMKU AM 01509 and ZMKU AM 01513), have a narrow light brown stripe on tibia. Pineal body not visible in one male specimen from Pattani Province, Thailand (THNHM 21248).

##### Distribution.

Based on a combination of the morphological and genetic studies of *F. cancrivora* large type (Kurniawan et al. 2010; 2011; 2014), the reported distribution of *F. raja* (Chan-ard 2013; Chuaynkern, and Chuaynkern 2012), and localities of specimens examined in this study, *F. cancrivora* is distributed from south of the Isthmus of Kra in Thailand, West Malaysia, Kalimantan (Borneo), Sumatra, West and Central Java, and Bali in Indonesia, with introduced populations in Papua New Guinea and Guam (Christy et al. 2007; Frost, 2019). In Thailand, *F. cancrivora* was confirmed to occur at Phatthalung, Nakhon Si Thammarat, Pattani, Songkhla, and Narathiwat Province (Fig. 1; Table 1).

##### Habitat, ecology and natural history.

**Figure 7.**
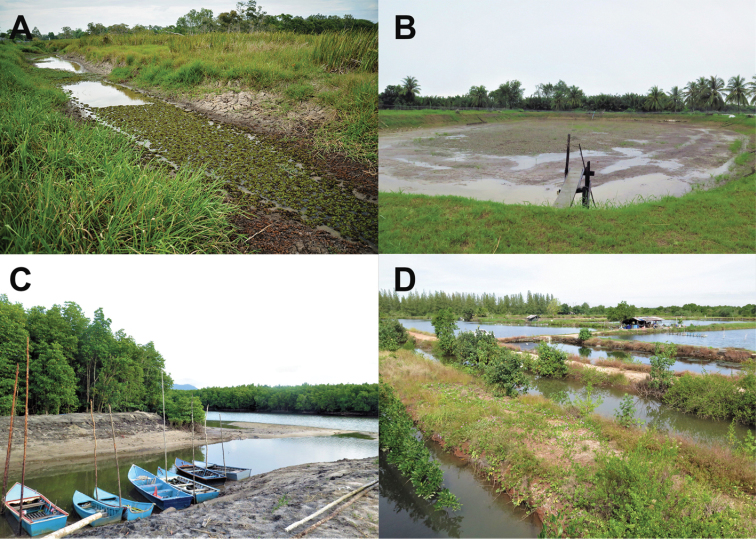
Exemplar habitats in Thailand of **A***Fejervarya cancrivora* at a wetland in Khuan Khanun District, Patthalung Province **B***F. cancrivora* at a brackish shrimp pond near Pak Phanang river, Pak Phanang District, Nakhon Si Thammarat Province **C***F. moodiei* at mangrove forest in Thai Mueang Distrinct, Phang-nga Province, and **D***F. moodiei* at brackish fish ponds near mangroves at the mouth of the Prasae River, Kleang District, Rayong Province. Photograph **A** by Attapol Rujirawan.

Specimens were collected in Thailand (Khuan Khanun District, Phatthalung Province and Pak Panang District, Nakhon Si Thammarat Province) at night (1900–2200 h) following light rain during May and October 2016. At Khuan Khanun, frogs were sampled in grasslands, rice paddy fields near standing or slow flowing ditches, and ponds at 1–24 m elevation (Fig. 7A). These were found sitting on the ground near water bodies, or hiding within grass or in mud cracks in the ground, and jumped to water bodies when disturbed. Other anuran species found in syntopy at this locality included *Duttaphrynus melanostictus* (Schneider, 1799), *F. limnocharis*, *Hoplobatrachus rugulosus* (Wiegmann, 1834), *Hylarana erythraea* (Schlegel, 1837), *Polypedates leucomystax* (Gravenhorst, 1829) and *Microhyla butleri* Boulenger, 1900. At Pak Phanang District, frogs were collected at night (1900–2100 h) after heavy rain in November 2017. These were found on the bank or in the water of brackish shrimp ponds near the Pak Phanang River at 0 m asl (Fig. 7B). No other anuran species were found in syntopy at this locality, although *F. moodiei* was sampled at a site approximately 4.5 air-km, or 5.2 km following the river course, upriver (below).

#### Fejervarya moodiei

Taxon classificationAnimaliaAnuraDicroglossidae

(Taylor, 1920)

C78704E6-2E13-55B1-B5ED-FF9B2CD1B950

Rana moodiei Taylor, 1920: 234
Rana cancrivora : Taylor 1962: 377
Fejervarya moodiei : Dubois and Ohler 2000: 35; Brown et al. 2013: 17
Fejervarya cancrivora : Chan-ard 2003: 107; Chuaynkern and Chuaynkern 2012: 169; Kurniawan et al. 2010: 3
Fejervarya Bangladesh mangrove type Islam et al. 2008: 1084
Fejervarya cancrivora mangrove type Kurniawan et al. 2010: 222; Kurniawan et al. 2011: 12
Fejervarya cf. cancrivora Harikrishnan & Vasudevan, 2018: 241

##### Diagnosis.

*Fejervarya moodiei* can be characterized by the following combination of characters: (1) medium to large size, SVL 42.7–62.7 mm in males, 50.0–81.8 mm in females (Table 4; Appendix 2, 3); (2) head length slightly greater than head width; (3) skin on dorsum and flank with spinules, and glandular warts, with irregular skin folds not arranged in series, with darker marking on dorsal surface of forelimbs and hindlimbs; (4) relative finger lengths II < IV < I < III; (5) Most individual have dermal fringe on fingers II and III; (6) prepollax indistinct; (7) palmar tubercles indistinct; (8) foot moderately webbed, with webbing formula I1–11/2II1–2III1–2IV2–1V; (9) dermal flap on postaxial side of Toe V; (10) Fejervaryan lines absent; (11) inner metatarsal tubercles prominent; (12) indistinct inner tarsal ridge on distal half to two-thirds of tarsus (Fig. 6C–D) and (13) vocal sacs in adult males with wrinkled skin covered by triangular, very dark brown blotches on each side of throat.

##### Description of holotype.

**Figure 8.**
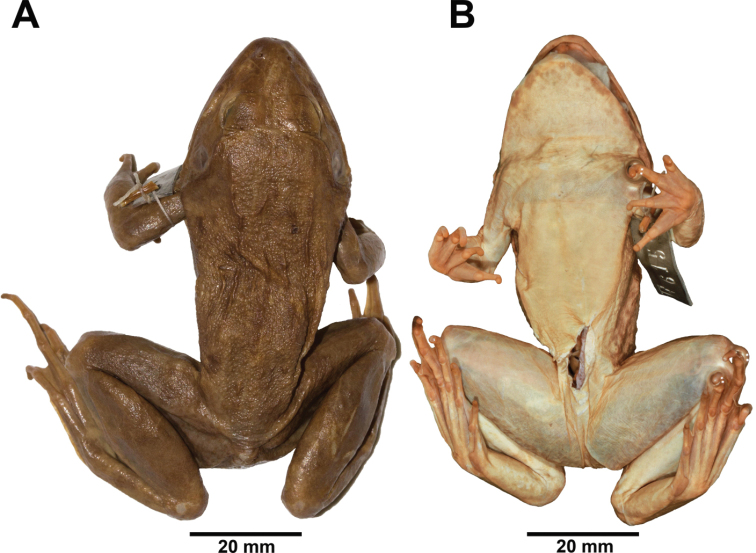
Adult female holotype of *Fejervarya moodiei* (CM 3724) in preservative in **A** dorsal and **B** ventral views. Photograph **B** by Carnegie Museum of Natural History.

Taylor (1920) described the species based on an adult female, CM 3724, from Manila, Luzon, Philippines (Fig. 8A, B; Appendix 3). We supplement his description of the holotype, as follows: rather large body size; head narrow, slightly longer than wide; snout tip oval in dorsal view, round in lateral view, projecting beyond lower jaw; nostril dorsolateral, oval, with small lateral flap, closer to tip of snout than eye; canthus indistinct, rounded; loreal region slightly concave and oblique [loreal region broadly sloping, not concave according to Taylor (1920)]; eye diameter about 60% snout length [eye diameter equal to snout length according to Taylor (1920)]; interorbital region flat, about half width of upper eyelid and slightly less than internarial distance; pineal body present; tympanum distinct, rounded, about 90% of eye diameter, not depressed relative to skin of temporal region, tympanic rim weakly elevated relative to tympanum, dorsoposterior margin obscured by supratympanic fold; vomerine ridge present in two strongly oblique series, very slightly closer to each other than to choanae [based on Taylor (1920), not examined by us].

Forelimbs short, rather robust; fingers rather long, slightly swollen; tips of fingers slightly rounded, terminus slightly swollen but not expanded into discs; relative finger lengths II < IV < I < III [first finger longer than second and fourth according to Taylor (1920)]; dermal fringe on fingers absent; webbing on fingers absent; subarticular tubercles distinct; supernumerary tubercles absent; prepollex indistinct, oval; palmar tubercles indistinct.

Hindlimbs moderately short, robust; tibia slightly longer than thigh, but shorter than distance from base of inner metatarsal tubercle to tip of Toe IV; toe long, stout; tips of toes rounded, not expanded into discs; relatively toe lengths I < II < III < IV, webbing moderate, deeply excised between toes, formula I1–11/2II1–1III1–2IV2–1V, Toe I webbed to base of distal phalanx; preaxial side of Toe II webbed to point between distal subarticular tubercle and distal phalanx, continuing as narrow fringe to base of distal phalanx; postaxial side of Toe II webbed to base of distal phalanx; preaxial side of Toe III webbed to distal subarticular tubercle, continuing as narrow fringe to base of distal phalanx, postaxial side of Toe III webbed to base of distal phalanx; preaxial side of Toe IV wedded to webbed to proximal distal subarticular tubercle, continuing as narrow fringe to base of distal phalanx, postaxial side of Toe IV wedded to webbed to proximal distal subarticular tubercle, continuing as narrow fringe to base of distal phalanx, Toe V webbed to base of distal phalanx; dermal flap well developed, extending along postaxial side of Toe V from level of inner metatarsal tubercles to distal phalanx; subarticular tubercles prominent, inner metatarsal tubercle prominent, oval, length about 30% that of Toe I; distinct dermal ridge extending along inner metatarsal tubercle to distal phalanx of Toe I; indistinct inner tarsal ridge on distal two-third of tarsus (Fig. 7C); outer metatarsal tubercles absent; supernumerary tubercles absent; tarsal tubercle absent.

Skin on snout and between the eyes shagreened; skin on eyelid shagreened with glandular warts; skin on dorsum shagreened with glandular warts and irregular skin folds; dorsolateral fold extending posteriorly to two-thirds length of dorsum; skin on side of head smooth; skin on flank with glandular warts; skin on cloacal region with glandular warts; forelimbs shagreened; thigh with indistinct glandular warts; tibia, tarsus, throat, chest and belly smooth.

##### Coloration of holotype in preservative.

Coloration mostly lost in preservative. Dorsum and side of head medium brown with a few dark brown markings; tympanum translucent brown with pale brown spot in center; flank pale brown with faint brown marbling; three wide brown vertical spots on upper lips; dorsal surfaces of forelimbs, thigh, tibia, and foot medium brown with a few dark brown spots, posterior surface of thigh with irregular pattern of indistinct dark brown marbling on light background; chin, chest, belly, and ventral surfaces of forelimb and hindlimb pale brown; ventral surfaces of hand and foot pale brown; lower lip pale brown with a few dark brown spots; vertebral and tibial stripes absent; Fejervaryan lines absent.

##### Coloration of referred Thai specimen in life.

**Figure 9.**
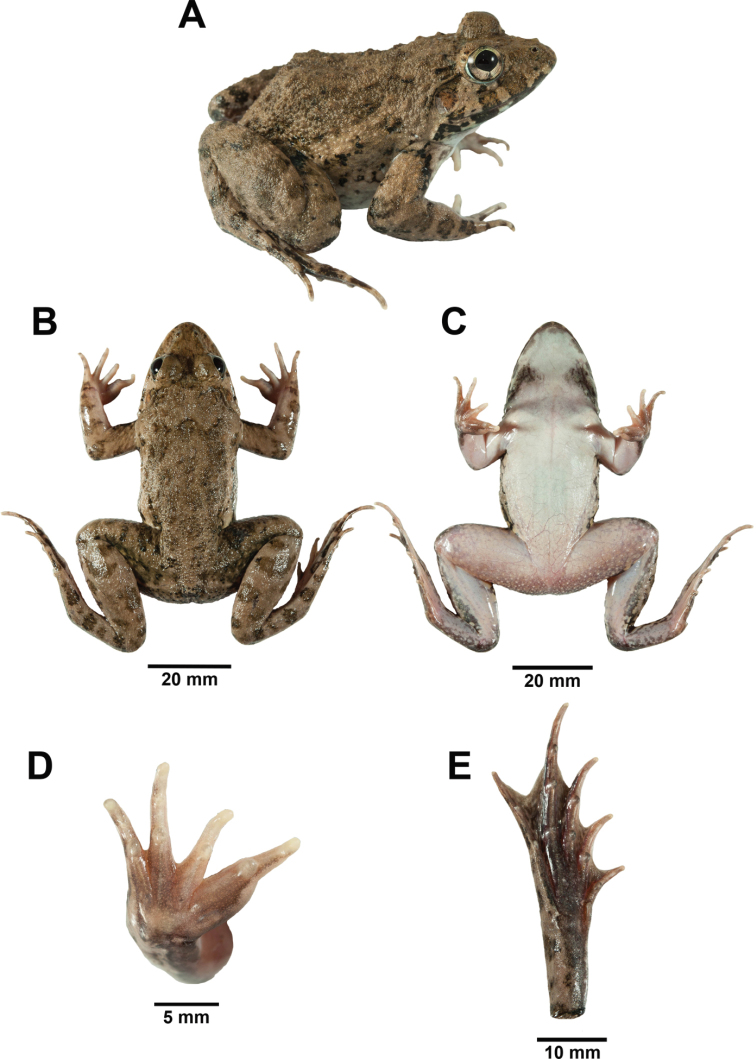
Adult male *Fejervarya moodiei* (ZMKU AM 01390) from Mueang Phang-nga District, Phang-nga Province, Thailand (SVL = 60.6 mm) immediately prior to preservation in **A** right lateral **B** dorsal **C** ventral **D** right palmar, and **E** right plantar views. Photographs by Attapol Rujirawan.

Adult male ZMKU AM 01390 (Fig. 9A–E) from Mueang Phang-nga District, Phang-nga Province, Thailand. SVL 44.7 mm. Dorsum and side of head light brown with indistinct olive brown marking; olive-brown band between outer margin of upper eyelids; tympanum with orange-brown blotches in center; olive-brown streak on canthus rostralis from tip of snout to eye; dark brown streak from eye along supratympanic fold to posterior rim of tympanum; flank creamy white with dark brown marbling; three wide dark brown spots on upper lips; dorsal part of limbs: forelimbs, thigh, tibia, and foot light brown with olive-brown transverse spots, posterior part of thigh with irregular pattern of dark brown marbling on creamy yellow background; ventral part of body: chin creamy white with indistinct mottled dark brown, triangular dark brown blotches and mottling on each side of throat; forelimbs, chest, belly creamy white and hindlimbs with indistinct dark brown mottling, hand brown and foot dark brown; lower lip creamy white with dark brown spots; Fejervaryan lines absent.

##### Variations.

Vomerine ridges slightly closer to choanae than to each other in some individuals. Most adult males have nuptial pads with small translucent spinules on dorsal and medial surface of Finger I from base of distal phalanx to base of prepollax, but some individuals have the nuptial pad extending to slightly over the base of prepollex. Most adult males have dense, fine spinules covering only the chin, but some individuals have dense, fine spinules on the chin and chest. Adult males have vocal sac present on each side of throat with wrinkled skin covered by triangular, very dark brown blotches. Adult males with larger spinules and glandular warts on dorsum, dorsal surfaces of forelimbs, flank, hindlimbs and vent region. Females are distinctly larger in size (Table 4, Appendix 3), lack nuptial pads and vocal sacs, having fewer spinules and glandular warts on dorsal surface of body and flank than males.

Dorsal coloration in preservative varies in males and females from brown to dark brown with darker markings. Two female specimens from Trat Province, Thailand (ZMKU AM 01444 and 01451) have dark orange markings on anterior part of dorsum. Markings or transverse spots on dorsum and dorsal surfaces of forelimbs and hindlimbs usually distinct, but faint in a few individuals. Coloration on flank usually creamy white, but pale brown, with dark brown marbling, in some individuals. Ventral coloration usually creamy white, but pale brown with indistinct dark mottling on chin and chest in some individuals. Hand usually creamy white, but light brown in some individuals. Most specimens have dermal fringe on fingers II and III (males *N* = 21, 70%; females *N* = 23, 69.7%), but some individuals lack this fringe (males *N* = 9, 30%; females *N* = 10, 30.3%). One specimen from Narathiwat Province, Thailand (THNHM 19720) has a vertebral stripe.

##### Distribution.

Based on a combination of morphological and genetic studies of *F. cancrivora* mangrove type (Kurniawan et al. 2010, 2011, 2014) and *Fejervarya* Bangladesh mangrove type (Islam et al. 2008), the reported distribution of *F. cancrivora* (Chan-ard 2003; Chuaynkern and Chuaynkern 2012), *F. moodiei* (Brown et al. 2013), and F. cf. cancrivora (Harikrishnan and Vasudevan 2018), and specimens studied here, *F. moodiei* occurs in coastal areas from eastern India, the Andaman and Nicobar Islands, and southern China, southward through Vietnam, Thailand, Myanmar, Malaysia and Luzon Island in the Philippines. In Thailand, *F. moodiei* was documented in all coastal regions except the extreme southeastern Gulf of Thailand coast, where it is replaced by *F. cancrivora* (Fig. 1).

##### Habitat, ecology, and natural history.

In Thailand, specimens were collected at night (1900–2200 h) in a variety of coastal habitats at elevations ranging from 0–16 m asl. Most specimens were observed in marshes near slow flowing ditches, ponds, or canals in mangrove forest (Fig. 7C). The species was also found in man-made environments such as agricultural fields adjacent to mangroves. In Kleang District, Rayong Province, most specimens were collected in and around brackish fish ponds and ditches in mangrove areas near the mouth of the Prasae River (Fig. 7D). Specimens from Pak Phanang District, Nakhon Si Thammarat Province were found around brackish shrimp ponds and ditches near the mouth of the Pak Phanang River. Frogs were observed sitting on the ground, under tree roots, or in or on the bank of water bodies. When disturbed, they usually escaped into holes in the ground or jumped into brackish water. No other anuran species were found in syntopy at this locality, although *F. cancrivora* was sampled at a site approximately 4.5 air-km, or 5.2 km following the river course, downriver (above).

##### Comparisons.

Twelve species of *Fejervarya* are known (Frost 2019), with nine species occurring in East and Southeast Asia (Sanchez et al. 2018). Four species of *Fejervarya* occur in Thailand, including *F. limnocharis* (Gravenhorst, 1829), *F. multistriata* (Hallowell, 1861), *F. orissaensis* (Dutta, 1997), and *F. triora* Stuart et al, 2006. Three additional *Fejervarya* species occur in adjacent countries, including *F. iskandari*Vieth et al. 2001, *F. sakishimensis*Matsui et al., 2007, and *F. kawamurai*Djong et al., 2011.

*Fejervarya cancrivora* and *F. moodiei* differ from all of these species by having the following combination of characters: (1) medium to large body size (vs. small to medium, SVL about 30–40 mm in males for *F. iskandari*, *F. kawamurai*, *F. limnocharis*, SVL about 40–55 mm in males for *F. multistriata*, *F. orissaensis*, *F. triora* [Dutta 1997; Matsui et al. 2007; Chuaynkern et al. 2009; Djong et al. 2011]; (2) webbing formula: I1–11/2II1–2III1–2IV2–1V (vs. I0–1II0–11/2III0–11/2IV11/2–0V in *F. vittigera*, I1–2II1–2III1–22/3IV22/3–11/2V in *F. limnocharis*, I1–2II1–2III1–22/3IV21/3–1V in *F. iskandari*, I1–2II1–2III11/2–22/3 IV22/3–1V in *F. multistriata*, I1–2II1–21/2III11/2–3IV3–11/2V in *F. sakishimensis*, I1–2II1–21/3III11/2–3IV3–1V in *F. kawamurai*); (3) having triangular or rectangular dark brown blotches covering vocal sacs on both sides of throat (vs. black “M” shape across throat in *F. kawamurai*, *F. limnocharis*, *F. sakishimensis*, *F. triora*, *F. vittigera*); (4) having prepollax indistinct (vs. distinct in *F. kawamurai*, *F. iskandari*, *F. limnocharis*, *F. sakishimensis*, *F. triora*), and (6) having palmar tubercles indistinct (vs. distinct *F. kawamurai*, *F. limnocharis*, *F. sakishimensis*, *F. triora*).

*Fejervarya moodiei* differs from *F. cancrivora* by having: (1) SVL 42.7–62.7 mm in males, 50.0–81.8 mm in females (vs. 60.2–79.8 mm in males, 85.1–107.1 mm in females of *F. cancrivora*, Table 4; Appendix 2, 3); (2) indistinct, slightly raised inner tarsal ridge on tarsus (vs. distinct, strongly raised inner tarsal ridge on distal half or two-thirds of tarsus in *F. cancrivora*) (Fig. 5A–D); and (3) in body proportions (Table 4). In Thailand, *F. moodiei* appears to be closely associated with brackish water in or adjacent to mangrove forest, whereas *F. cancrivora* also occurs in freshwater wetlands.

## Discussion

Our study clarifies that two species of crab-eating frogs (*Fejervarya cancrivora* complex) occur in mainland Southeast Asia: *F. moodiei* in coastal regions throughout mainland Southeast Asia, with replacement by *F. cancrivora* sensu stricto in extreme southern Thailand (on the Gulf of Thailand coast) and peninsular Malaysia. These findings corroborate those of Kurniawan et al. (2010; 2011) that the name *F. moodiei* is the correct name to apply to populations of the *F. cancrivora* complex throughout most of coastal mainland Southeast Asia. Our study provides the first molecular evidence that *F. raja* from southern Thailand represents only a large-bodied population of *F. cancrivora* sensu stricto, as suspected but untested by Iskandar (1998) and Kurniawan et al. (2010, 2011). Both *F. cancrivora* and *F. moodiei* have wide geographic distributions that span coastlines of both mainland and insular Southeast Asia (Fig. 1), a likely testament to their remarkable tolerance of salt and brackish water (e.g., Gordon et al. 1961; Balinsky et al. 1972; Wright et al. 2004; Hopkins and Brodie 2015). Although our findings of two Southeast Asian frog species having wide geographic distributions is inconsistent with many recent analyses of other taxa (e.g., Stuart et al. 2006; Aowphol et al. 2013; Geissler et al. 2014; Phimmachak et al. 2015; Wogan et al. 2016; Sheridan and Stuart 2018), the conserved morphology of the *F. cancrivora* complex has long hindered accurately understanding species diversity and distributions of these frogs, as evidenced by the conflicting interpretations of experienced systematic herpetologists (e.g., Smith 1930; Inger 1954; Taylor 1962). Hence, the integrative taxonomic approach used here that incorporated both molecular and morphological data, including from topotypes and name-bearing type specimens, respectively, proved to be imperative for resolving these uncertainties.

This study provides a basis for revising the identifications of historical and contemporary records (both museum vouchers and literature descriptions) of crab-eating frogs to improve the finer-scale details of the geographic ranges, as well as the natural histories, of *F. cancrivora* and *F. moodiei* in mainland Southeast Asia. Our sampling did not reveal *F. cancrivora* and *F. moodiei* to occur in sympatry, but did find the two species to occur in shrimp ponds that were separated by only approximately 4.5 air-km (or 5.2 km following the river course) along the Pak Phanang River in Pak Phanang District, Nakhon Si Thammarat Province, Thailand (Fig. 1; Appendix 1). The Pak Phanang locality of *F. moodiei* (8°19.850'N, 100°11.870'E) lies closer to the river mouth and has higher saltwater intrusion than does the Pak Phanang locality of *F. cancrivora* (8°17.454'N, 100°11.229'E) that lies further upstream of a complex system of water gates and irrigation canals that were constructed in the 1960s to reduce saltwater intrusion and facilitate rice production (Boromthanarat et al. 1991). It is not known if the two species were separated at these shrimp ponds because the two localities are coincident with the boundaries of their geographic ranges, or if the two species differ in saltwater tolerance and other aspects of their ecology. Future sampling to clarify the fine-scale partitioning of the two species where their ranges come into contact is warranted.

## Supplementary Material

XML Treatment for Fejervarya cancrivora

XML Treatment for Fejervarya moodiei

## Appendix 1

Specimens examined.

*Fejervarya cancrivora* (Gravenhorst, 1829): **Indonesia**, Java: CNHM 131093, 131100, 131105–09, 161102, 191098, 313095, FMNH 131108, 131111; West Java, Cianjur FMNH 256688 (neotype); **Thailand**, Nakhon Si Thammarat Province: FMNH 174052–53; Pak Phanang District: (8°17.454'N, 100°11.229'E) ZMKU AM 01507–13; THNHM 25499; Narathiwat Province, Su-Ngai Kolok District: THNHM 19221, 20754; Tak Bai District: THNHM 19722–23, 19726–28, 19764–71; Pattani Province, Nong Chick District: THNHM 15623, 21248–49; Phatthalung Province: CNHM 175923–26; Khuan Khanun District: (7°45.580'N, 100°9.446'E): ZMKU AM 01415–26, 01427–29, (7°44.127'N, 100°8.635E) ZMKU AM 01430–34; Pak Phayun District: THNHM 19852–57; Songkhla Province: THNHM 04332, 04955–56

*Fejervarya moodiei* (Taylor, 1920): **Malaysia**: CNHM 161312; **Philippines**, Northern Luzon: FMNH 161693, 161697; Luzon, Manila: CM 3724 (holotype); **Thailand**: Chonburi Province: FNHM 190532, THNHM 04919–21, Mueang Chonburi District: THNHM 06408–12; Chumphon Province, Moo Ko Chumphon National Park: THNHM 01030–33; Krabi Province, Ko Lanta District (7°35.702'N, 99°4.272'E): ZMKU AM 01405–14, Mueang Krabi District (8°4.502'N, 98°55.506'E): ZMKU AM 01487–90; Nakhon Si Thammarat Province, Khanom District (9°12.760'N, 99°50.969'E): ZMKU AM 01435–41; Pak Phanang District (8°19.850'N, 100°11.870'E): ZMKU AM 01464–79; Narathiwat Province, Tak Bai District: THNHM 19720–21, 19724–25; Phang-nga Province, Mueang Phang-nga District (8°25.998'N, 98°30.973'E): ZMKU AM 01390–98; Phatthalung Province, Songkhla lake: THNHM 04332–33; Phuket Province, Mueang Phuket District (7°54.522'N, 98°24.425'E): ZMKU AM 01376–83, 01399–404; Prachuap Khiri Khan Province, Kui Buri District (12°8.143'N, 99°57.737'E): ZMKU AM 01491–92, Sam Roi Yot District: ZMKU AM 01368–71; Ranong Province, Kra Buri District (10°19.435'N, 98°45.894'E): ZMKU AM 01372–75, 01480–86, Suk Samran District: THNHM 25736; 26002, 26016; Rayong Province, Klaeng District THNHM 14252–64, (12°42.164'N, 101°41.634'E): ZMKU AM 01514–20; Samut Prakarn Province, Phra Pradaeng District, Bang Krachao Sub-district: THNHM 26075–78; Satun Province, La-ngu District (6°51.861'N, 99°45.484'E): ZMKU AM 01493–506; Songkhla Province, Songkhla lake: THNHM 02403–05; Surat Thani Province, Ko Samui District (9°33.220'N, 100°3.327'E): ZMKU AM 01384–89; Mueang Surat Thani District, Makham Tia Sub-district: THNHM 05857–58; Trat Province, Ko Chang District (12°0.178'N, 102°22.639'E): ZMKU AM 01442–63; Klong Yai District: THNHM14292–94; Mueang Trat District: THNHM 16631–36, 24452; Trang Province, Kantang District, Ko Libong: THNHM 02249.

## Appendix 2.

**Table:** Morphological measurements (mm) of adult male specimens of *Fejervarya*. Data are given as mean and standard deviation, followed by range in parentheses.

Characters	*F. cancrivora* neotype	*F. cancrivora* Indonesia and Malaysia	*F. cancrivora* (previously *F. raja*) Thailand	*F. moodiei* (previously *F. cancrivora*) Thailand
	*N* = 1	*N* = 4	*N* = 26	*N* = 30
SVL	66.9	74.6 ± 3.8 (71.4 – 79.8)	71.0 ± 5.7 (60.2–78.9)	51.4 ± 5.4 (42.7–62.7)
HL	25.6	29.7 ± 1.1 (28.6 – 31.0)	29.1 ± 2.1 (24.5–32.4)	20.5 ± 1.8 (17.3–25.0)
HW	23.7	26.2 ± 1.6 (24.37 – 27.9)	26.6 ± 2.3 (22.0–30.5)	17.7 ± 1.8 (14.4–22.1)
STL	19.1	22.0 ± 0.6 (21.3– 22.8)	21.56 ± 1.4 (18.4–23.7)	15.4 ±1.4 (13.3–18.8)
NS	4.3	4.9 ± 0.2 (4.7–5.2)	5.2 ± 0.4 (4.5–6.0)	3.8 ± 0.40 (3.1–4.7)
SL	10.5	11.7 ± 0.7 (11.0–12.6)	12.2 ± 0.7 (10.8–14.1)	8.4 ± 0.8 (6.8–10.2)
NTL	14.8	17.1 ± 0.78 (16.2–18.1)	16.6 ± 1.2 (14.2– 18.2)	12.0 ± 1.0 (10.5 –14.4)
EN	6.0	6.9 ± 0.5 (6.6–7.6)	6.7 ± 0.5 (5.6–7.6)	4.5 ± 0.4 (3.6–5.2)
TEL	2.3	3.2 ± 0.2 (3.08 – 3.4)	2.7 ± 0.6 (1.9–3.7)	1.7 ± 0.4 (1.2–2.8)
TD	5.1	5.2 ± 0.5 (4.6–5.7)	5.1 ± 0.4 (4.2–6.0)	4.1 ± 0.4 (3.3–4.9)
IN	2.8	3.9 ± 0.1 (3.8–3.9)	3.5 ± 0.4 (2.9–4.4)	2.5 ± 0.4 (1.9–3.5)
EL	6.6	6.9 ± 0.6 (6.1–7.4)	7.0 ± 0.7 (5.5–8.6)	5.9 ± 0.6 (4.8–7.1)
IOD	3.0	3.4 ± 0.2 (3.1–3.6)	3.5 ± 0.5 (2.7– 4.4)	3.2 ± 0.4 (2.5–3.8)
UEW	4.9	6.4 ± 0.4 (6.07 – 6.83)	5.9 ± 0.7 (4.9–7.3)	4.2 ± 0.5 (3.2–5.1)
HAL	16.2	18.00 ± 0.9 (16.9–19.1)	17.5 ± 1.2 (15.5–19.5)	12.7 ± 1.4 (10.6–15.7)
FAL	13.1	15.0 ± 0.9 (14.1–16.0)	13.6 ± 0.9 (11.5–15.0)	10.2 ± 1.2 (8.2–12.2)
THIGHL	30.2	34.7 ± 2.8 (31.6–38.0)	34.1 ± 2.8 (29.1–39.1)	23.4 ± 2.7 (19.0–29.3)
TL	34.5	38.4 ± 2.1 (36.5–41.4)	37.0 ± 3.0 (30.7 – 42.7)	24.44 ± 2.73 (19.84 – 30.3)
FOL	37.2	39.8 ± 1.0 (38.6–40.7)	38.35 ± 2.5 (30.7–43.3)	26.6 ± 3.1 (21.5–32.9)
TFOL	56.3	59.8 ± 1.4 (58.4–61.3)	56.2 ± 4.1 (48.7–62.8)	38.6 ± 4.6 (30.4–48.1)
1FL	13.8	14.3 ± 0.8 (13.3–15.0)	13.3 ± 1.1 (11.6–16.1)	9.4 ± 1.3 (7.2–12.4)
IMTL	4.1	3.9 ± 0.5 (3.5– 4.5)	4.3 ± 0.4 (3.2–5.2)	3.0 ± 0.5 (1.9–4.0)
ITL	12.4	13.2 ± 1.3 (11.3–14.3)	13.3 ± 1.0 (11.9–14.9)	9.2 ± 1.4 (7.0–11.3)
HL/HW	1.1	1.1 ± 0.0 (1.1–1.2)	1.1 ± 0.0 (1.0–1.2)	1.2 ± 0.0 (1.1–1.2)
IOD/HW	0.1	0.1 ± 0.0 (0.1–0.1)	0.1 ± 0.0 (0.1–0.2)	0.2 ± 0.2 (0.1–0.2)
SL/HL	0.4	0.4 ± 0.1 (0.4–0.4)	0.4 ± 0.0 (0.4– 0.5)	0.4 ± 0.0 (0.4–0.5)
EL/HL	0.3	0.2± 0.0 (0.2–0.3)	0.2 ± 0.0 (0.2–0.3)	0.3 ± 0.0 (0.2–0.3)
NS/EN	0.7	0.72 ± 0.1 (0.6–0.8)	0.8 ± 0.1 (0.7–0.9)	0.8 ± 0.1 (0.7–1.0)
EL/SL	0.6	0.6 ± 0.0 (0.6–0.6)	0.6 ± 0.0 (0.5–0.7)	0.7 ± 0.1 (0.6–0.8)
EL/EN	1.1	0.0 ± 0.1 (0.9–1.1)	1.0 ± 0.1 (1.0–1.2)	1.3 ± 0.2 (1.0–1.6)
IN/IOD	0.9	1.1 ± 0.1 (1.1–1.3)	1.0 ± 0.2 (0.8–1.5)	0.8 ± 0.1 (0.5–1.2)
TD/EL	0.8	0.8 ± 0.1 (0.6–0.8)	0.7 ± 0.1 (0.6–0.9)	0.7 ± 0.1 (0.6–0.8)
TEL/EL	0.3	0.5 ± 0.1 (0.4–0.6)	0.4 ± 0.1 (0.3–0.5)	0.3 ± 0.1 (0.2–0.4)
FAL/HAL	0.8	0.9 ± 0.0 (0.9–1.0)	0.8 ± 0.0 (0.7–0.9)	0.8 ± 0.1 (0.7– 0.9)
THIGHL/TL	0.9	0.8 ± 0.0 (0.8–0.9)	0.8 ± 0.0 (0.7–0.9)	1.0 ± 0.0 (0.9–1.1)
FOL/TL	1.1	1.0 ± 0.0 (1.0–1.1)	1.0 ± 0.0 (1.0–1.1)	1.1 ± 0.1 (0.9–1.2)
IMTL/TL	0.1	0.1 ± 0.0 (0.1–0.1)	0.1 ± 0.0 (0.1–1.1)	0.2 ± 0.0 (0.9–0.2)

## Appendix 3.

**Table:** Morphological measurements (mm) of adult female specimens of *Fejervarya*. Data are given as mean and standard deviation, followed by range in parentheses.

**Character**	***F. moodiei***	***F. cancrivora***	***F. cancrivora***	***F. moodiei***
	CM 3724 Holotype	Indonesia and Malaysia	(previously *F. raja*)	(previously *F. cancrivora*)
			Thailand	Thailand
	*N* = 1	*N* = 2	*N* = 12	*N* = 32
SVL	73.3	93.9 ± 7.0	95.5 ± 3.5	69.0 ± 10.1
		(93.0–98.0)	(107.1–85.1)	(50.0–81.8)
HL	29.8	35.3 ± 1.1	37.5 ± 2.0	27.1 ± 4.0
		(34.5–36.1)	(34.2–41.6)	(19.2–33.0)
HW	27.3	28.0 ± 1.1	36.2 ± 2.3	24.5 ± 4.1
		(27.2–28.7)	(31.8–39.4)	(17.2–30.9)
STL	22.4	35.1 ± 1.1	28.1 ± 1.4	20.1 ± 2.8
		(34.3–35.9)	(25.5–29.9)	(14.6–23.6)
NS	5.3	5.9 ± 0.3	6.9 ± 0.6	4.9 ± 0.7
		(5.7–6.1)	(5.8–8.1)	(3.5–6.1)
SL	11.4	15.2 ± 0.3	16.3 ± 1.0	11.1 ± 1.6
		(14.9–15.4)	(14.8–18.3)	(7.9–13.2)
NTL	17.1	22.1 ± 0.8	21.5 ± 1.4	15.6 ± 2.2
		(21.5–22.6)	(19.0–24.1)	(11.3–18.2)
EN	6.2	8.6 ± 0.3	9.0 ± 0.7	6.0 ± 0.8
		(8.4–8.8)	(7.9–10.4)	(4.3–7.2)
TEL	3.8	5.2 ± 0.9	4.5 ± 0.8	3.0 ± 0.8
		(4.6–5.9)	(3.6–6.1)	(1.4–4.0)
TD	5.8	6.4 ± 0.6	6.5 ± 0.6	4.9 ± 0.6
		(5.6–6.8)	(5.7–7.6)	(3.8–5.7)
IN	3.3	4.1 ± 0.2	4.7 ± 0.6	3.2 ± 0.5
		(4.0–4.2)	(3.9–5.7)	(2.2–4.0)
EL	6.7	7.5 ± 0.2	8.5 ± 0.7	6.9 ± 0.7
		(7.4–7.6)	(7.7–9.7)	(5.4–8.4)
IOD	3.1	4.4 ± 0.5	4.8 ± 0.5	3.9 ± 0.64
		(4.0–4.8)	(4.0–5.7)	(2.9–5.4)
UEW	5.9	7.4 ± 0.4	7.5 ± 0.7	5.4 ± 0.8
		(7.1–7.7)	(6.3–8.4)	(4.2–6.9)
HAL	17.0	22.5 ± 1.5	22.5 ± 1.9	16.5 ± 2.3
		(23.4–21.5)	(19.6–26.1)	(12.0–19.6)
FAL	12.9	17.6 ± 0.3	17.6 ± 1.4	13.0 ± 2.2
		(17.4–17.8)	(15.4–20.1)	(9.2–16.4)
THIGHL	34.4	40.5 ± 4.6	43.8 ± 2.8	29.9 ± 4.3
		(37.2–43.7)	(40.0–48.8)	(21.6–35.8)
TL	35.7	46.4 ± 0.5	48.0 ± 4.1	31.9 ± 4.5
		(46.0–46.7)	(42.6–54.9)	(23.1–37.0)
FOL	40.2	48.5 ± 0.7	49.2 ± 3.9	34.8 ± 5.0
		(48.0–49.0)	(43.6–547)	(24.6–42.7)
TFOL	57.2	73.5 ± 1.7	73.1 ± 7.7	49.9 ± 7.0
		(72.3–74.7)	(64.2–86.2)	(35.4–58.8)
1FL	13.6	18.2 ± 0.6	17.8 ± 1.4	13.0 ± 2.1
		(17.7–18.6)	(15.2–20.3)	(9.2–15.8)
IMTL	4.4	4.9 ± 0.5	5.7 ± 0.5	4.1 ± 0.7
		(4.6–5.3)	(4.9–6.4)	(2.9–5.4)
ITL	14.8	16.7 ± 0.2	17.5 ± 1.9	12.4 ± 1.8
		(16.5–16.9)	(13.8–20.5)	(8.5–15.2)
