# Corrigenda: Species delimitation of crab-eating frogs (*Fejervarya cancrivora* complex) clarifies taxonomy and geographic distributions in mainland Southeast Asia. ZooKeys 883: 119–153. https://doi.org/10.3897/zookeys.883.37544

**DOI:** 10.3897/zookeys.897.48818

**Published:** 2019-12-09

**Authors:** Siriporn Yodthong, Bryan L. Stuart, Anchalee Aowphol

**Affiliations:** 1 Department of Zoology, Faculty of Science, Kasetsart University, Bangkok, Thailand Kasetsart University Bangkok Thailand; 2 Section of Research & Collections, North Carolina Museum of Natural Sciences, Raleigh, NC, USA North Carolina Museum of Natural Sciences Raleigh United States of America

## Abstract

NA

In our recent publication (Yodthong et al. 2019), there are errors in Table 1 (Column: GenBank accession numbers). The corrections are as follows:

**Page 125**:

Accession number of ZMKU AM 01442: “MN453508” should be “MN453513”

Accession number of ZMKU AM 01446: “MN453509” should be “MN453514”

Accession number of ZMKU AM 01451: “MN453510” should be “MN453515”

Accession number of ZMKU AM 01467: “MN453511” should be “MN453516”

Accession number of ZMKU AM 01475: “MN453512” should be “MN453517”

Accession number of ZMKU AM 01479: “MN453513” should be “MN453518”

Accession number of ZMKU AM 01485: “MN453514” should be “MN453519”

Accession number of ZMKU AM 01493: “MN453515” should be “MN453520”

Accession number of ZMKU AM 01498: “MN453516” should be “MN453521”

Accession number of ZMKU AM 01503: “MN453517” should be “MN453522”

Accession number of ZMKU AM 01516: “MN453518” should be “MN453526”

Accession number of ZMKU AM 01520: “MN453519” should be “MN453527”

**Page 126**:

Accession number of ZMKU AM 01418: “MN453520” should be “MN453508”

Accession number of ZMKU AM 01423: “MN453521” should be “MN453509”

Accession number of ZMKU AM 01425: “MN453522” should be “MN453510”

Accession number of ZMKU AM 01426: “MN453523” should be “MN453511”

**Page 127**:

Accession number of ZMKU AM 01430: “MN453524” should be “MN453512”

Accession number of ZMKU AM 01507: “MN453525” should be “MN453523”

Accession number of ZMKU AM 01509: “MN453526” should be “MN453524”

Accession number of ZMKU AM 01511: “MN453527” should be “MN453525”

